# Identification of core genes for early diagnosis and the EMT modulation of ovarian serous cancer by bioinformatics perspective

**DOI:** 10.18632/aging.202524

**Published:** 2021-01-25

**Authors:** Yanna Zhang, Xun Wang, Xiancheng Chen

**Affiliations:** 1 State Key Laboratory of Biotherapy, Collaborative Innovation Center for Biotherapy, West China Hospital, Sichuan University, High Technological Development Zone, Chengdu 610041, Sichuan, People’s Republic of China

**Keywords:** ovarian serous cancer, early diagnosis, biomarkers, EMT

## Abstract

Ovarian serous carcinoma (OSC), as a common malignant tumor, poses a serious threat to women's health in that epithelial-mesenchymal transformation (EMT)-related modulation becomes heavily implicated in the invasion and progression of OSC. In this study, two core genes (BUB1B and NDC80) among the 16 hub genes have been identified to be involved in the molecular regulation of EMT and associated with the poor early survival of OSC at stages I+II. Through the Gene Regulatory Networks (GRN) analysis of 15 EMT regulators and core genes, it was revealed that TFAP2A and hsa-miR-655 could elaborately modulate EMT development of OSC. Next genetic variation analysis indicated that EMT regulator ELF3 would also serve as a crucial part in the occurrence and progression of OSC. Eventually, survival investigation suggested that TFAP2A, ELF3 and hsa-miR-655 were significantly associated with the overall survival of progressive OSC patients. Thus, combined with diversified bioinformatic analyses, BUB1B, NDC80, TFAP2A, ELF3 and hsa-miR-655 may act as the key biomarkers for early clinical diagnosis and prognosis evaluation of OSC patients as well as potential therapeutic target-points.

## INTRODUCTION

Ovarian cancer, about 90% originated from ovarian surface epithelium, is one of the most common malignant tumors in the female reproductive system, with a very low five-year survival rate and the highest mortality rate among all kinds of female tumors [1]. The molecular mechanisms in ovarian cancer development, recurrence and metastasis are complex and changeable, leading to insufficient innovation in early clinical diagnosis and treatment models [2]. As the most common pathological type among ovarian carcinomas, ovarian serous cancer (OSC) accounts for 80–95% of ovarian malignancies [3]. Although current therapeutic strategies for OSC have improved significantly, the 5-year survival rate of OSC is still much lower than other gynecological malignancies, with a relapsing rate of ~ 70% of patients [4]. Elucidating the molecular mechanism of OSC may help us understand the pathogenesis and progress of OSC and identify new targets for effective treatment. However, relatively little is known about the molecular events leading to the development of this highly invasive disease [5].

Epithelial-mesenchymal transformation (EMT), one of the core biological process in the occurrence and development of epithelial ovarian cancer (EOC), has been considered as the crucial mechanism of ovarian surface epithelial cells participating in ovarian tumor growth, migration, invasion, metastasis and drug resistance formation [6]. Moreover, it is necessary to explore more meaningful biomarkers for early clinical diagnosis and EMT biological mechanism related to the pathogenesis, early prevention and treatment of OSC.

The main features of EMT are loss of epithelial phenotype and acquisition of stromal features [7], which makes epithelial cells lose intercellular connection, reduce adhesion, and obtain mesenchymal characteristics [8]. The dissociation of cell connections during the EMT process is not a "collapse" caused by simple cytoskeletal changes, but a more fine-tuned way in that first is the dissociation of adhesive connections, then the cytoskeletal changes and the multi-step dissociation of tight connections process [9]. EMT plays an essential role in wound healing, stemness acquirement, tissue fibrosis and in OSC deterioration covering cancer invasiveness progression, distant metastasis, relapse and drug resistance developments [10]. Previous studies have shown that EMT transcriptional regulators, such as CDH1/E-cadherin, CDH2/N-cadherin, ZEB1, ZEB2, SNAI1, SNAI2, TWIST1 and TWIST2, are essential for promoting cell invasion, migration, proliferation and angiogenesis [11, 12]. During EMT conversion, tumor cells undergo obvious cytoskeletal reconstruction based on the expression of various transcription factors and activation of surface receptors related to EMT phenotype [13]. Dr. Ruby Huang's research indicated that AXL, when activated as an EMT regulator, could interact with other proteins, thus forming an intracellular signal to enhance the invasion, migration and proliferation of ovarian cancer cells as the crucial signal of EMT promoting cancer development [14]. ARK5/NUAK1 and HOXA10, as the regulatory factors in EMT cascade loop, were remarkably upregulated, when compared with adjacent normal tissues, thus enhancing invasiveness of ovarian cancer [15]. In the main signaling pathways closely related to EMT, BIRC5, CTNNB1 and relevant other proteins could also enhance the migration and proliferation of ovarian cancer cells with the expression of EMT markers [16, 17]. Meanwhile, PARP-1, also as a core EMT regulator, could play an important role in OSC progression [18, 19]. However, another EMT regulator ELF3, when up-regulated, could mediate the EMT signal molecules cascade to increase the expression of epithelial markers and decrease the mesenchymal markers in ovarian cancer [20]. Therefore, EMT is closely involved in the process of tumor development, invasion, metastasis and recurrence of OSC [13]. Nevertheless, the relation of expression profile of transcription factors and proteins associated with EMT to the diverse pathological features of OSC has not been comprehensively investigated.

In this study, the obvious common differential expression genes (co-DEGs) from the gene expression data of 3 OSC datasets in the Gene Expression Omnibus (GEO) database were screened for Gene Ontology (GO) and Kyoto Encyclopedia of Gene and Genome (KEGG) functional enrichment analysis. Through the protein-protein interactions (PPIs) network co-expression interaction of co-DEGs, we carried out the integrated bioinformatics analysis to find those core genes with a significant hint for early clinical diagnosis on OSC. Then, co-expression analyses for core genes and 15 EMT regulators involved in the multiple pathological features of ovarian cancer were conducted to detect the critical regulatory role of these genes and regulators in the occurrence and progression of OSC. Next, ovarian specific co-expression regulation analysis, KEGG function enrichment analysis, genetic variation, mutation count, overall survival status, and GRN analysis would be managed respectively. In summary, based on comprehensive bioinformatics analyses [21–23], this study would assist with exploring the potential biomarkers, elucidating the mechanisms underlying relevant pathophysiological events and finally exploiting effective and reliable targeted therapies for OSC.

## RESULTS

### DEGs identification with data normalization

**Figure 1:**
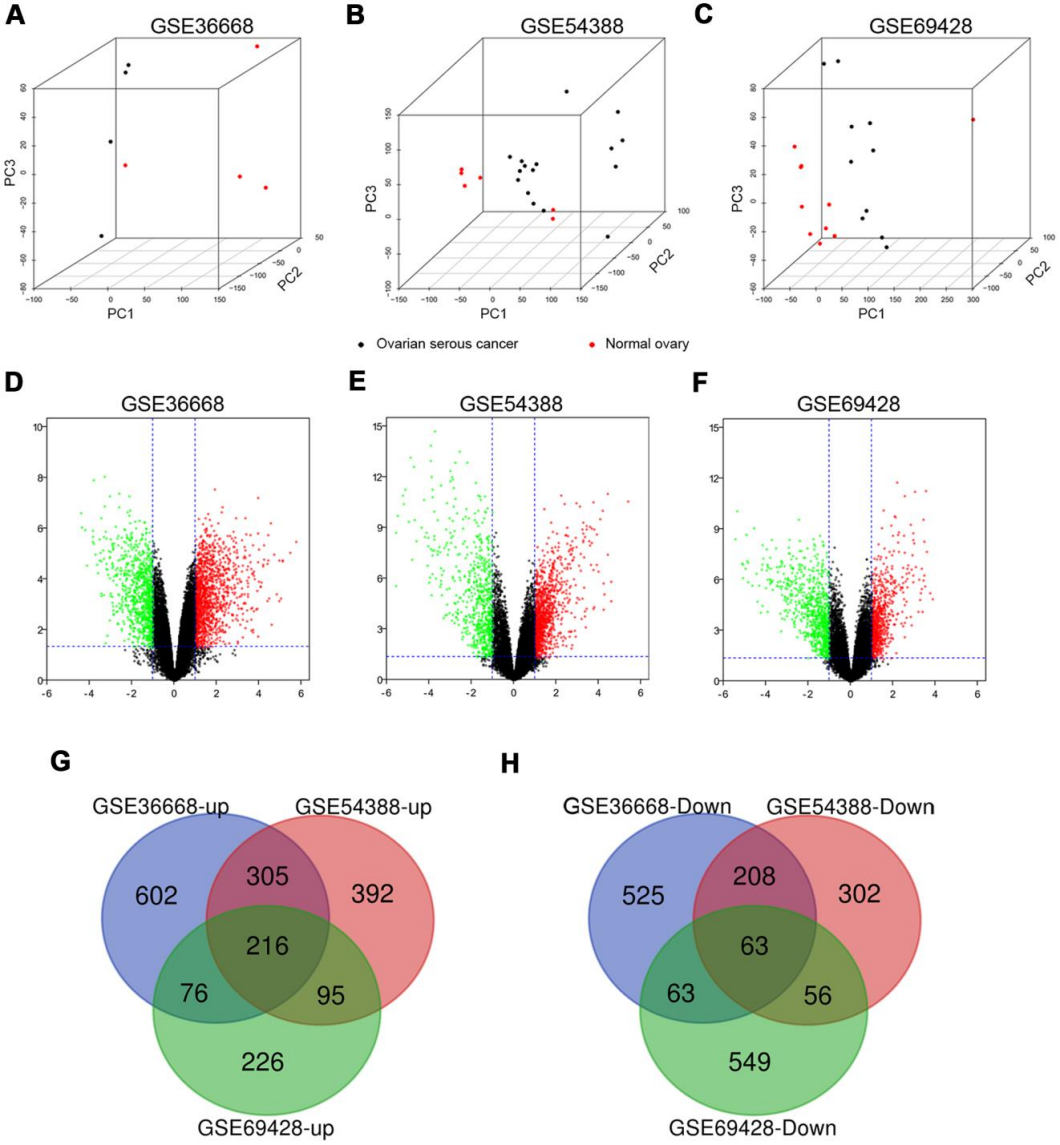
**The distribution of expression situation and DEGs identification among GSE36668, GSE54388 and GSE69428 after normalization.** (**A**–**C**) Whole transcriptomes were subjected to PCA on expressed genes to assess sample diversity and relatedness between OSC tissues (black dot) and normal ovary tissues (red dot). See also Supplementary Videos 1–3 (Supporting Information). (**D**, **E**) Volcano plots represent DEGs between OSC tissues and normal ovary tissues. Red dots indicate upregulation in DEG (LogFC≥1, *p*-value<0,05), and green dots indicate down regulation (LogFC≤-1, *p*-value<0.05). Three-way Venn diagram based on whole transcriptomes represents the distribution of the up expressed genes (**G**) and the down expressed genes (**H**) among these datasets.

Three expression profiles (GSE36668, GSE54388, and GSE69428) were obtained from the GEO database, and the specific details were listed in Supplementary Table 1. These datasets, covering OSC tissues and normal ovary tissues, were both from patients with OSC, with GSE36668 including 4 OSC tissues and 4 normal ovary tissues, GSE54388 containing 16 OSC tissues and 6 normal ovary tissues, GSE69428 consisting of 10 OSC tissues and 10 normal ovary tissues (Supplementary Table 1). We evaluated these datasets by Principal component analysis (PCA) after data normalization (Figure 1A–1C and Supplementary Videos 1–3). Then, the heatmaps of gene expression in GSE36668, GSE54388 and GSE69428 were shown in Supplementary Figure 2A–2C. After gene annotation, the DEGs were screened in each data series with Log FC≥1 or Log FC≤-1 and *p*-value<0.05 as the criteria for selection. The GSE36668 dataset included 2058 DEGs, covering 1199 upregulated and 859 downregulated genes (Figure 1D); the GSE54388 dataset included 1637 DEGs consisting of 1008 upregulated and 629 downregulated genes (Figure 1E); the GSE69428 dataset included 1344 DEGs consisting of 613 upregulated and 731 downregulated genes (Figure 1F) as shown in Volcano plots. The details of significant DEGs from each dataset were displayed in Supplementary Table 2. Moreover, the overlap of co-DEGs in three datasets contained 279 genes, as Venn diagrams showed, consisting of 216 upregulated co-DEGs (Figure 1G) and 63 downregulated co-DEGs (Figure 1H) when compared with normal ovary samples.

### Functional annotation and PPIs network of co-DEGs

**Figure 2:**
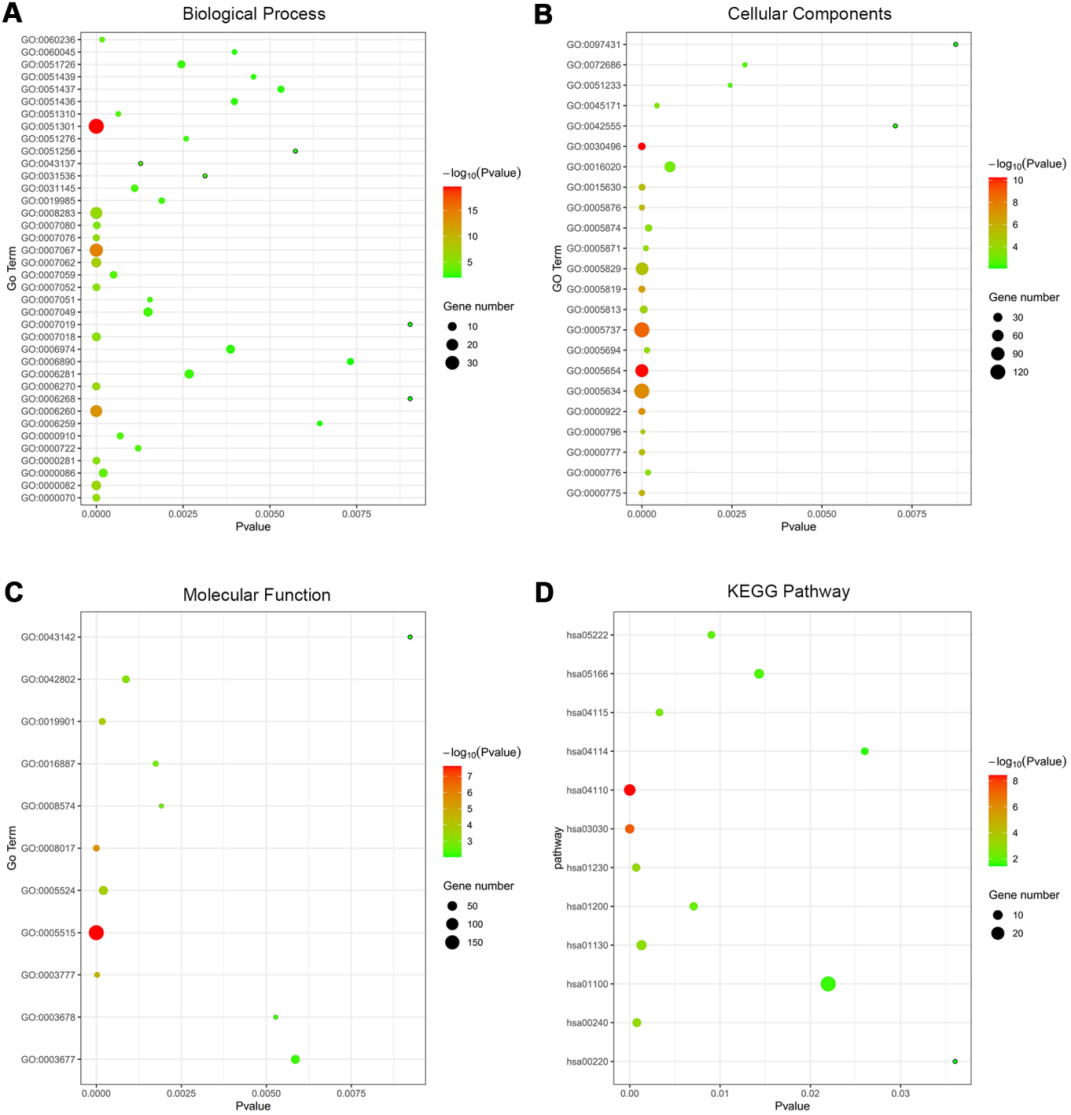
**Functional annotation of co-DEGs between OSC tissues and normal ovary tissues using GO terms of BP, CC, MF and KEGG pathway.** Bubble map for Go terms of BP (**A**), CC (**B**), MF (**C**) and KEGG pathway (**D**).

279 co-DEGs were subjected to GO enrichment for biological process (BP), molecular function (MF) and cellular component (CC) analyses according to criterion of *p*-value<0.01. The BP analysis of co-DEGs was mainly focused on the cell division (GO:0051301), mitotic nuclear division (GO:0007067) and DNA replication (GO:0006260) (Supplementary Table 3 and Figure 2A). For CC analysis, the co-DEGs were notably enriched in the midbody (GO:0030496), nucleoplasm (GO:0005654) and cytoplasm (GO:0005737) (Supplementary Table 3 and Figure 2B). Concerning the MF analysis, the co-DEGs were mostly enriched in protein binding (GO:0005515), microtubule binding (GO:0008017) and microtubule motor activity (GO:0003777) (Supplementary Table 3 and Figure 2C). In addition, we utilized the DAVID to categorize co-DEGs in the KEGG database. Subsequent results indicated that the co-DEGs were significantly involved in 12 signaling pathways, such as cell cycle (hsa04110), DNA replication (hsa03030), and biosynthesis of amino acids (hsa01230) (Supplementary Table 3 and Figure 2D).

**Figure 3:**
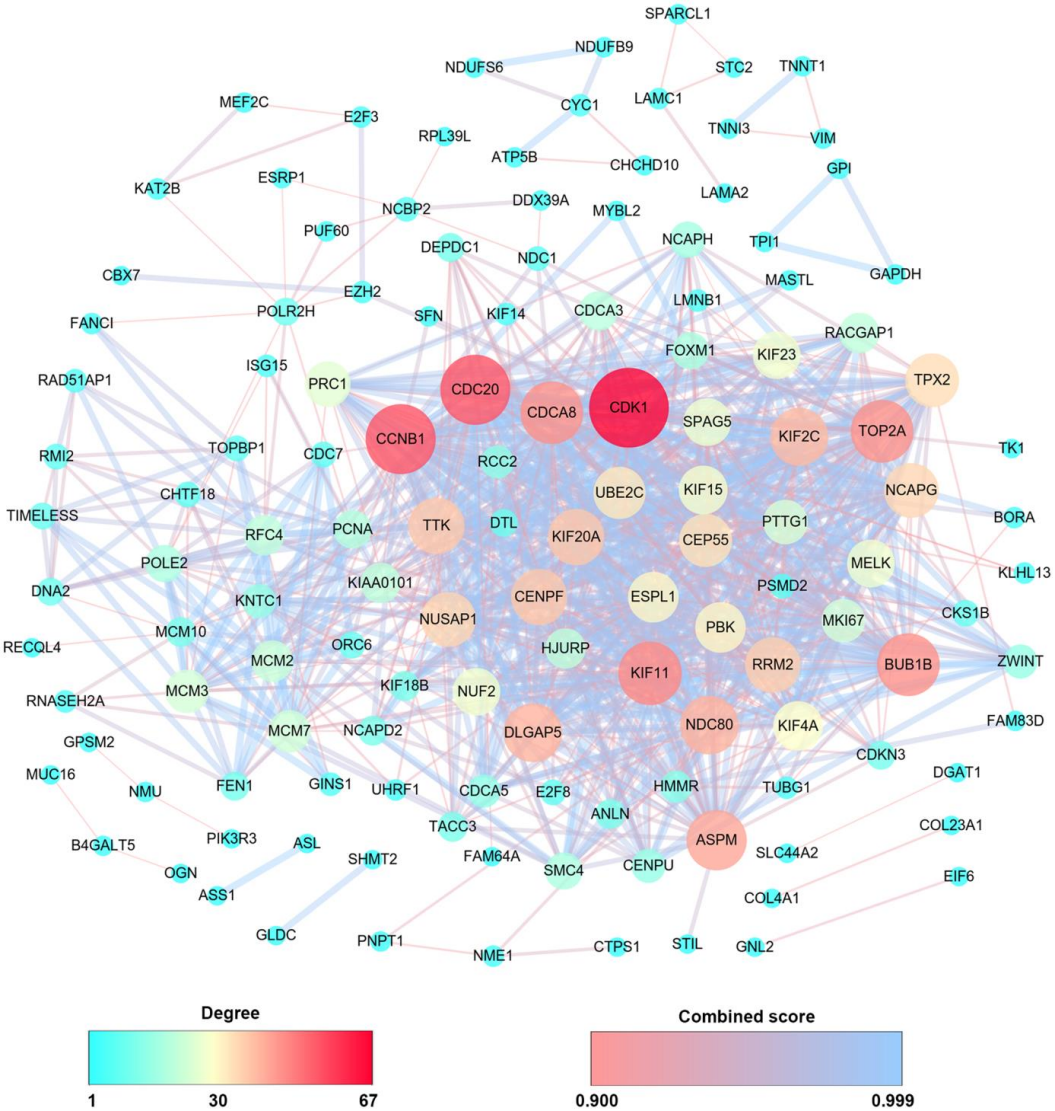
**PPIs co-expression network from 279 co-DEGs.** The sphere color and size represent the degree of nodes with the line color indicating the combined score among them.

**Figure 4:**
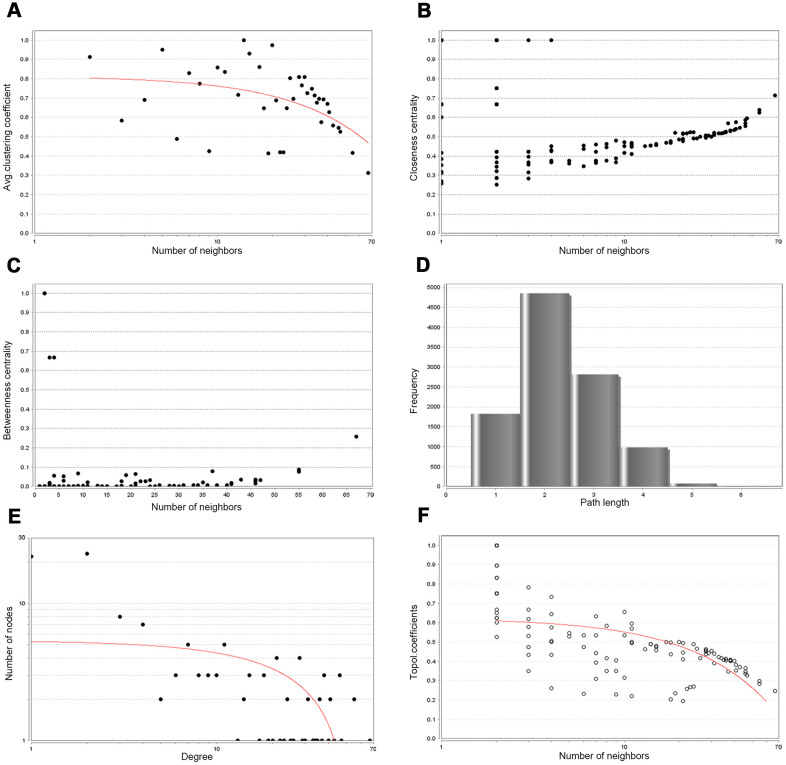
**Backbone network PPIs topology parameters.** (**A**) Avg. clustering coefficient. (**B**) Closeness centrality. (**C**) Betweenness centrality. (**D**) Shortest path length distribution. (**E**) Distribution of the node degree. (**F**) Topology coefficient.

Then, PPIs network was constructed to explore the interaction relationship among co-DEGs in the pathogenesis of OSC. The interaction score≥0.09 (high-confidence interaction score) for nodes was considered as a pronounced PPIs network (Figure 3). The backbone network of co-DEGs consists of 134 nodes with an estimated clustering coefficient of 0.671 and PPIs enrichment (*p*-value<1.0e-16). Moreover, the topological parameters of co-DEGs PPIs network were displayed in Table 1, including the Avg. clustering coefficient (Figure 4A), closeness centrality (Figure 4B), betweenness centrality (Figure 4C), shortest path length distribution (Figure 4D), the distribution of the node degree (Figure 4E) and topological coefficients (Figure 4F).

**Table 1:** Topological parameters for co-DEGs PPIs network.

**Topological parameters**	**Comprehended values**
Number of nodes	134
Clustering co-efficient	0.617
Network density	0.103
Network heterogeneity	1.098
Network centralization	0.407
Shortest paths	10572(59%)
Characteristic path length	2.303
Avg. number of neighbors	13.642

### Core genes identification

Next, Degree>40 was set as criterion to screen hub genes. Simultaneously, based on the module analysis, nine significant modules were obtained (Supplementary Table 4). The seed nodes in these nine modules were also regarded as hub genes. According to the cut-off criteria, we collected 16 hub genes totally (Supplementary Table 5). Overall survival impacts of hub genes on patients with OSC at all stages were performed by Kaplan-Meier plotter (Supplementary Figure 3). A survival forest map for hub genes was shown as Supplementary Figure 3A and the hub genes-related survival curves were presented in Supplementary Figure 3B–3Q. As a result, we discovered 13 hub genes which were significantly associated with the overall survival of OSC patients among 16 hub genes, except for VIM [HR=0.92 (0.79-1.08), logrank *P*=0.3] (Supplementary Figure 3C), SPARCL1 [HR=1.15 (0.96-1.37), logrank *P*=0.13] (Supplementary Figure 3E), and CDCA8 [HR=1.14 (0.97-1.34), logrank *P*=0.13] (Supplementary Figure 3N). Additionally, the expression levels of the hub genes in different pathological stages of ovary cancer samples were displayed in Supplementary Figure 4. The results indicated that among different pathological stages there had been notable alterations in the expression levels for NDC80 [Pr(>F)=0.0496] (Supplementary Figure 4F), MCM2 [Pr(>F)=0.000134] (Supplementary Figure 4G), KAT2B [Pr(>F)=0.0365] (Supplementary Figure 4I), CHTF18 [Pr(>F)=0.000685] (Supplementary Figure 4K), and BUB1B [Pr(>F)=0.00954] (Supplementary Figure 4P). The dynamic overall trends revealed that the expression of above hub genes decreased gradually with the continuous progression of ovary cancer (Supplementary Figure 4). Then, the GEPIA2 database was used to verify the expression levels of BUB1B, CHTF18, KAT2B, MCM2 and NDC80 (Supplementary Figure 5). As shown in Supplementary Figure 5, except for CDCA8 (Supplementary Figure 5E), the expression levels of the other genes (Supplementary Figure 5A–5D) were statistically significant between ovary cancer tissues and normal ovary tissue from TCGA (The Cancer Genome Atlas) and GTEx (the genotype-tissue expression) data. Consequently, BUB1B, MCM2, KAT2B and NDC80 were identified as the key genes in diagnosis and prognosis of OSC patients.

**Figure 5:**
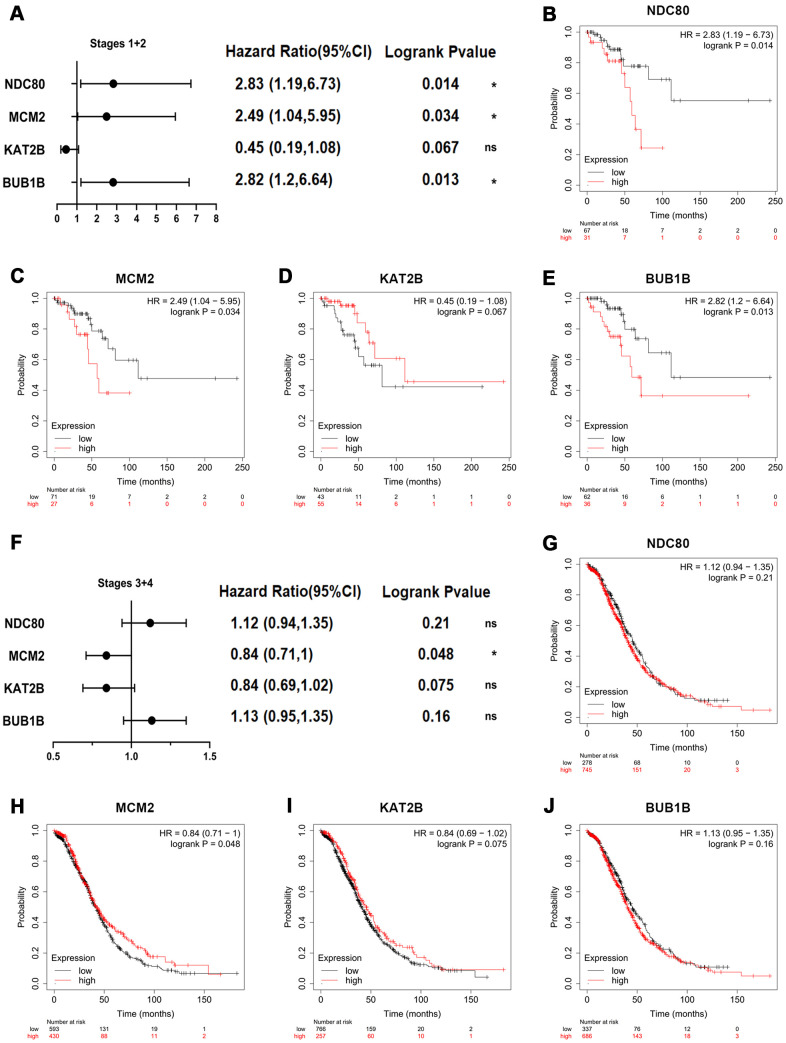
**Overall survival impact of key genes on patients with OSC at different stages.** (**A**) Survival prognosis forest map related to key genes at early stages (stages I+II) in OSC patients. Each point in the forest plot represents the Hazard ratio (HR) of the gene, and the lines on both sides of the point represent the 95% confidence interval (95%CI). Survival curves were constructed by the Kaplan-Meier plotter based on the low and high expression of the key genes in OSC patients, including (**B**) NDC80, (**C**) MCM2, (**D**) KAT2B and (**E**) BUB1B. (**F**) Survival prognosis forest map related to key genes at advanced stages (stages III+IV) in OSC patients. Kaplan-Meier overall survival analysis for OSC patients with the expression of key genes, covering (**G**) NDC80, (**H**) MCM2, (**I**) KAT2B and (**J**) BUB1B. Logrank *p*-value<0.05 was considered statistically significant.

Then, the prognostic impact information of key genes on patients with OSC at different stages was explored by Kaplan-Meier plotter database (Figure 5). The key genes-related survival forest map at early stages (stages I+II) was shown in Figure 5A and relevant survival curves were respectively presented in Figure 5B–5E. The higher expression levels of NDC80 [HR=2.83 (1.19-6.73), logrank *P*=0.014] (Figure 5B), MCM2 [HR=2.49 (1.04-5.95), logrank *P*=0.034] (Figure 5C) and BUB1B [HR=2.82 (1.2-6.64), logrank *P*=0.013] (Figure 5E) were notably related to poor overall survival at early stages in OSC patients. Meanwhile, the key genes-related survival forest map at advanced stages (stages III+IV) was shown in Figure 5F and relevant survival curves were respectively presented in Figure 5G–5J, indicating that only one of four key genes was notably associated with the overall survival of OSC patients at advanced stages (Figure 5G–5J), in that high expression of MCM2 [HR=0.84 (0.71-1), logrank *P*=0.048] (Figure 5H) was associated with improved overall survival in OSC patients at advanced stages. In summary, BUB1B and NDC80 were deemed as the core genes for early diagnosis of patients with OSC.

### Association of expression patterns of core genes with EMT regulators

**Figure 6:**
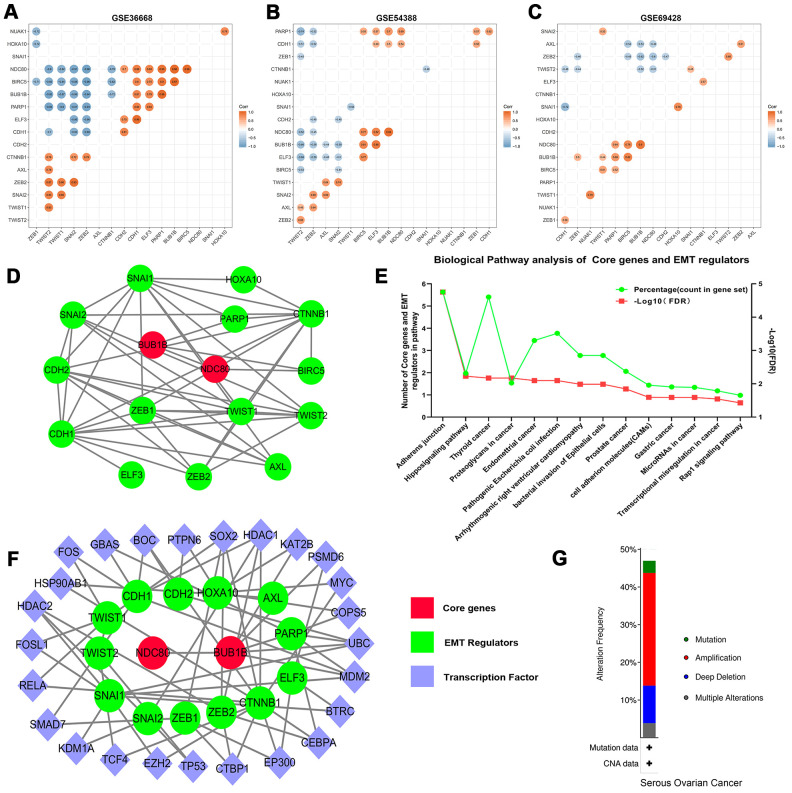
Correlation analysis of the core genes and EMT regulators in datasets (**A**) GES36668, (**B**) GES54388, (**C**) GSE69428. (**D**) The PPIs network of core genes and EMT regulators. The red color indicates core genes, and the green color predicts EMT regulators. (**E**) KEGG pathway enrichment analysis of core genes and EMT regulators. Only the enriched pathways with FDR<0.05 were presented. The green lines represent percentage of count in gene set and the red lines represent -Log10 (FDR). (**F**) The ovary-specific PPIs integrated network of core genes and EMT regulators, the red color indicates core genes, the green color predicts EMT regulators and the purple color represents transcription factors. (**G**) An overview of the alteration of the core genes and EMT regulators in the genomics datasets of OSC in the TCGA database.

The heatmap of expression levels of core genes and EMT regulators in GEO datasets revealed that the expressions of core genes and EMT regulators have exhibited significantly differences between OSC tissues and normal ovary tissues (Supplementary Figure 6). Further analyses indicated that correlation between core genes and EMT regulators were statistically significant (Figure 6A–6C, *P*<0.05). And then STRING analyses for co-expression of core genes and EMT regulators verified that the core genes were closely related to the expression modulation of EMT regulators (Figure 6D). Meanwhile, functional KEGG enrichment analysis revealed that core genes and EMT regulators were mainly associated with adherens junction (hsa04520), Hippo signaling pathway (hsa04390), and Thyroid cancer (hsa05216) (Figure 6E and Supplementary Table 6). Furthermore, another online instrument NetworkAnalyst for ovary-specific PPIs network could also make out the notable roles of transcriptional regulation of core genes and EMT regulators in the development of ovarian cancer (Figure 6F).

### Characteristic alteration of core genes and EMT regulators

The cBioPortal assay validated that variation, mutation count of the 2 core genes and 15 EMT regulators related to overall survival status were notably altered in 146 (47%) of queried patients or samples. Alterations in ELF3, including amplification and missense mutations (Supplementary Figure 7), were most often (8%) among them. Relevant gene amplification was accounted for the highest percentage among the different types of mutation, gene amplification, deep deletion and multiple alterations (Figure 6G).

### GRN analysis for TF, miRNA, core genes and EMT regulators

**Figure 7:**
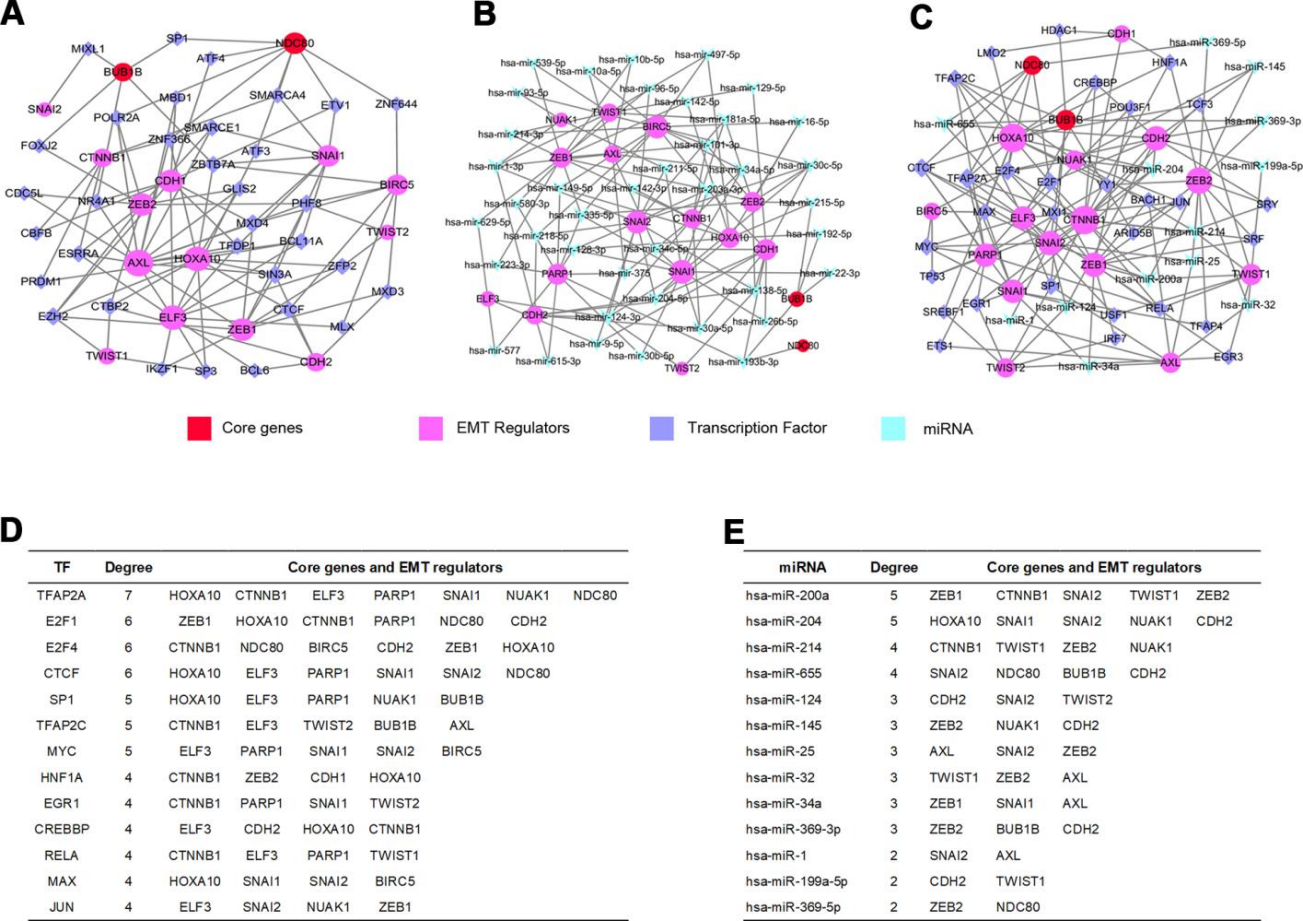
**GRN analysis of core genes and EMT regulators.** (**A**) Network of TFs-core genes and EMT regulators was obtained from ENCODE database. (**B**) Network of miRNAs-core genes and EMT regulators was obtained from TarBase and miRTarBase database. (**C**) Integrative regulatory network of TFs-miRNAs-core genes and EMT regulators. (**D**) Core genes and EMT regulators regulated by TFs. (**E**) Core genes and EMT regulators modulated by miRNAs.

In order to further confirm the main functions of core genes and EMT regulators, the potential modulation relationship among core genes, EMT regulators and TFs were discriminated based on TF and gene target data derived from the ENCODE ChIP-seq data (Figure 7A). Concurrently, a regulatory network among core genes, EMT regulators and miRNAs was compiled from the miRNA-gene interaction data collected from TarBase and miRTarBase (Figure 7B).

### TF-miRNA coregulatory interaction network

We constructed a TFs-miRNAs coregulatory network by collecting regulatory interaction information from the RegNetwork repository, containing 2 core genes, 15 EMT regulators, 30 TFs and 13 miRNAs with 152 edges (Figure 7C). The above analysis discovered that the transcription factor TFAP2A could monitor one core gene interacting with six EMT regulators (Figure 7D), and hsa-miR-655 could regulate two core genes (Figure 7E) among them.

### Core genes, EMT regulators, miRNA and TF validation

**Figure 8:**
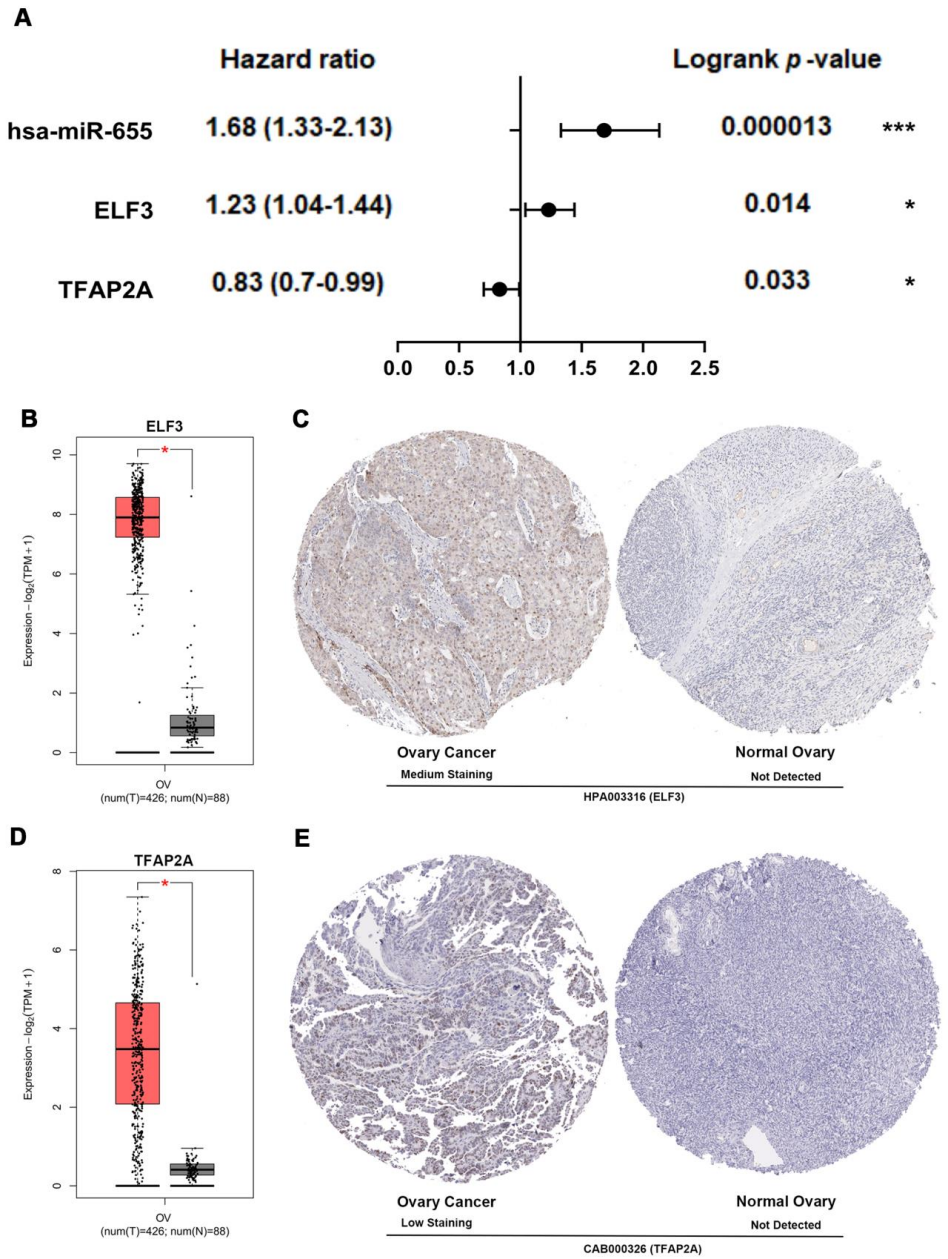
**Multidimensional validation and efficacy evaluation with the core TF, EMT regulator and miRNA.** (**A**) Survival prognosis forest map related to TFAP2A, hsa-miR-655 and ELF3 in patients with OSC. (**B**) The expression level of ELF3 from ovary cancer samples (red) and normal ovary samples (gray). (**C**) Validation of ELF3 from the HPA database. (**D**) The expression level of TFAP2A from ovary cancer samples and normal ovary samples. (**E**) Validation of TFAP2A from the HPA database.

The expression of core genes including BUB1B (Supplementary Figure 8A) and NDC80 (Supplementary Figure 8B) were significantly increased in OSC patients among GSE36668, GSE54388 and GSE69428 datasets. BUB1B (Supplementary Figure 9A) and NDC80 (Supplementary Figure 9C) were validated at a transcription level in multiple cancer types based on the Oncomine database in that BUB1B (Median rank 132, *p*-value=1.54e-06) (Supplementary Figure 9B) and NDC80 (Median rank 192.5, *p*-value=4.04e-08) (Supplementary Figure 9D) were highly expressed in ovary cancer samples compared with normal ovary tissues. As for prognostic value of core TF, EMT regulator and miRNA, high expression of TFAP2A [HR=0.83[0.7-0.99], logrank *P*=0.033] was associated with the improved overall survival in OSC patients by Kaplan-Meier plotter (Figure 8A). However, the high expression of hsa-miR-655 [HR=1.68[1.33-2.13], logrank *P*=0.000013] and ELF3 [HR=1.23[1.04-1.44], logrank *P*=0.014] was linked with worse overall survival in OSC patients (Figure 8A). The expression levels of the EMT regulator and core TF were presented based on TCGA and GTEx data (Figure 8B, 8D), with consistent expression trend in three datasets (Supplementary Figure 8C, 8D). Moreover, the protein levels of EIF3 (Figure 8C) and TFAP2A (Figure 8E) were significantly higher in ovary cancer tissues than in normal ovary tissues based on HPA database. Furthermore, in the CCLE database, the expression levels of BUB1B (Supplementary Figure 10A), NDC80 (Supplementary Figure 10B), ELF3 (Supplementary Figure 10C), TFAP2A (Supplementary Figure 10D) and hsa-miR-655 (Supplementary Figure 11A) were confirmed among different ovarian cancer cell lines. Additional qRT-PCR also detected the consistent expression trend in SKOV3 cell line with CCLE (Supplementary Figure 10E–10H). Then, we explored the decreased expression of hsa-miR-655 and increased expression of has-miR-200a in ovarian cancer from the miRCancer database. At the same time, compared with normal blood samples, we also found the expression of hsa-miR-655 decreased and hsa-miR-200a increased in blood samples of patients with ovarian cancer (Supplementary Figure 11B, 11C and Supplementary Table 8).

All above-mentioned observations confirmed that BUB1B and NDC80 may be used as the key biomarkers at early stages for patients with OSC, and TFAP2A, ELF3 and hsa-miR-655 could play crucial roles in the EMT occurrence and pathological prognostic factors for OSC patients.

## DISCUSSION

Since the ovary is located deep in the pelvis, about 70% of ovarian cancer cases have reached the advanced stage when they get diagnosed and distant metastases have already occurred, leading to its mortality rate ranking first among all kinds of gynecological malignant tumors [3]. Most patients with ovarian cancers may relapse after surgery or first line chemotherapy, and sometimes even after second line chemotherapy due to the ability of ovarian cancer stem cells to escape from these therapies or due to the reduced host immunosurveillance [24, 25]. Among them, OSC, as a common EOC and one of the most lethal gynecological tumors, accounts for 80–95% of ovarian malignancies [26]. Therefore, it is important to find reliable tumor biomarkers and explore the precise molecular mechanism of OSC for early clinical diagnosis, treatment and prognosis [27]. In recent years, with the help of large-scale screening and the rapid development of bioinformatics, hundreds of genes alterations have been revealed to be closely related to the diagnosis, therapy and prognosis of tumors [22, 28, 29].

In our study, three datasets for OSC samples, namely GSE36668, GSE54388 and GSE69428, were selected and downloaded from GEO database. Then we set up comprehensively bioinformatic scheme to perform co-DEGs, GO and KEGG pathway functional enrichment, PPIs network and overall survival analysis at different pathological stages. Through the PPIs network regulation of co-DEGs and the prognostic investigation of the hub genes-related survival rates at different pathological stages, we identified two core genes (BUB1B and NDC80) associated with the early diagnosis and prognosis of OSC. One of them, BUB1B, a key component of the mitotic checkpoint complex, is localized to the kinetochore and plays a pivotal role in inhibiting anaphase-promoting complex/cyclosome (APC/C), delaying the onset of anaphase and ensuring proper chromosome segregation. In addition, BUB1B was also remarkably enriched in cell cycle and in Human T-cell leukemia virus (HTLV)-1 infection on KEGG pathway database [30, 31]. Another core gene, NDC80, encodes a component of the NDC80 kinetochore complex, which consists of a N-terminal microtubule binding domain and a C-terminal coiled-coiled domain able to interact with other components of the complex. Its molecular functions are to organize and stabilize microtubule-kinetochore interactions for proper chromosome segregation. And NDC80 was notably enriched in cell cycle, covering overall mitotic phase at the metaphase, anaphase, and prometaphase transition [32, 33], with impairment function of mitotic spindle checkpoint found in many types of cancer. Moreover, emerging evidence also suggests that dysregulation of cell cycle signaling cross-cascade is commonly been observed in a broad range of human cancers [34, 35]. Especially, relevant co-DEGs were also most striking in cell cycle pathway associated highly with the tumorigenesis and progression of ovary cancer [36, 37].

Since the EMT plays a complex role in tumor metastasis and recurrence by enhancing cell invasion and migration or other possible accesses [38], it is crucial to elucidate molecular mechanisms regulating EMT biological events for improving treatment of patients with OSC [11]. Consequently, we screened 15 EMT regulatory factors involved closely in the diverse pathological features of ovarian cancer. Then, we explored gene alteration characteristics and GRN of two core genes and 15 EMT regulators. Subsequently, it was demonstrated that the EMT regulator ELF3 was the most frequent in genetic variation, thus eliciting the development and relapse of OSC. Meanwhile, in the GRN analysis for core genes and EMT regulators, it was also found that TFAP2A and hsa-miR-655 could exert a crucial function in the EMT modulation of ovarian cancer.

What was also intriguing was that TFAP2A could modulate the expression of target EMT regulator ELF3, positively, via interacting with their transcription start site (TSS) through the TF-miRNA comprehensive regulation analysis. Simultaneously, it has been demonstrated that TFAP2A could influence the transcription of target genes involved in many different types of cancer [39]. For example, relevant studies indicated that TFAP2A promotes the proliferation, migration and invasion of breast cancer cells [40], and Shi et.al revealed that TFAP2A promotes nasopharyngeal carcinoma cell proliferation and inhibits apoptosis [41]. Furthermore, microRNAs, as endogenous transcripts with almost 22 nucleotides in length, are considered as possible causative agents in cancer [42]. However, previous studies have identified hsa-miR-655 as a novel EMT-suppressive microRNA [43, 44]. Additionally, there is emerging evidence that women with endometriosis have a higher risk of developing ovarian cancer due to original disease progression and malignant transformation. Namely, endometriosis may be one of the main causes of ovarian cancer [45].

Overall, by integrating multiple microarrays of gene expression profiles, BUB1B and NDC80 have been identified to be vital in early stages for OSC development. TF-miRNA comprehensive investigations for core genes and EMT regulators elucidated that TFAP2A, hsa-miR-655/200a and ELF3 could exert crucial function and prognostic potential in the development and progression of OSC. Eventually, our investigation into bioinformatics for the core genes related to EMT biological process in OSC may bring an unusual perspective for the early diagnosis and prognosis evaluation of patients with OSC.

## CONCLUSIONS

In summary, BUB1B and NDC80 activation could play a pivotal role in the occurrence and development of OSC at stages I+II, and thus might serve as early clinical diagnosis biomarkers for patients with OSC. Furthermore, the gene variation and GRN analysis revealed that ELF3, TFAP2A and hsa-miR-655/200a could collectively coordinate BUB1B and NDC80 to modulate EMT biological process on the development and progression of OSC, which may serve as the potential therapeutic target-points.

## MATERIALS AND METHODS

### Microarray data collection

The raw expression profiles of GSE36668 [46], GSE54388 [20] and GSE69428 [47] were downloaded from GEO database (https://www.ncbi.nlm.nih.gov/geo/) based on microarray platform GPL570 (Affymetrix Human Genome U133 Plus 2.0Array). Details of each microarray data were provided in Supplementary Table 1. A microRNA expression profile (GSE31568) of blood was collected from ovarian cancer patients [48]. The dataset GSE31568 based on the platform of GPL9040 (febit Homo Sapiens miRBase 13.0) containing 15 samples with ovarian cancer and 70 samples without cancer was also downloaded from GEO database.

### Data preprocessing and Differential Expression Genes (DEGs) analysis

All data were normalized using NormalizeBetweenArray function from R package ‘LIMMA’ of the bioconductor project [49]. Data before and after normalization were shown in Supplementary Figure 1A–1C respectively. Next, we performed differential genes analyses (LogFC≥1 or LogFC≤-1, adjusted *p* value<0.05) by comparing OSC with normal ovary using ‘LIMMA’ R package.

### Screening co-DEGs and construction the PPIs network

The selected DEGs were separately uploaded to an online tool (http://bioinformatics.psb.ugent.be/webtools/Venn/), which could identify the co-DEGs among GSE36668, GSE54388 and GSE69428 datasets. Then, we utilized the online database STRING (Version 11.0, https://string-db.org/) to visualize the PPIs among co-DEGs [50]. To avoid an inaccurate PPIs network, we used a cutoff ≥0.9 (high-confidence interaction score) to obtain the striking PPIs and visualized in Cytoscape Version 3.6.1 [51]. Next, these most significant modules in the PPIs network were screened using MCODE, a package of Cytoscape, which could identify clusters in large protein networks according to the topology to build significant function modules. The criteria for selection included 5 aspects: MCODE score>5, node score cut-off=0.2, degree cut-off=2, Max depth=100, and k-score=2.

### Analyzing the backbone network

The NetworkAnalyzer package in Cytoscape was utilized to explore the topological parameters and centrality measures such as the Avg. clustering coefficient, distribution of the node degree, topological coefficients, shortest path length distribution, betweenness centrality, and closeness centrality for directed and undirected networks of co-DEGs PPIs backbone network.

### Functional enrichment analyses of co-DEGs

The co-DEGs were further analyzed via the Database for Annotation, Visualization and Integrated Discovery (DAVID, version 6.8, https://david.ncifcrf.gov/) to perform the GO and KEGG [52, 53]. The R ggplot2 package was adopted to visualize these data.

### Hub genes identification and analysis

The hub genes were selected for degree>40 nodes or seed genes of PPIs network significant function modules. In order to prove the hub genes related to OSC prognosis, overall survival analyses were performed using Kaplan-Meier Plotter (http://kmplot.com/analysis/). Patients with OSC were categorized into high-expression group and low-expression group, according to the expression of specific genes. The overall survival related to hub genes was analyzed for OSC patients at all stages in above 2 groups. The analysis results were visualized in the forms of survival prognosis forest map and survival curves. Logrank *p*-value<0.05 was regarded as statistically significant. Then, Gene Expression Profiling Interactive Analysis (GEPIA2, http://gepia2.cancer-pku.cn/#index) was performed to explore the alteration among ovary cancer samples at different pathological stages. ANOVA was accomplished to evaluate the statistical significance of variations. Pr(>F)<0.05 was regarded as statistically significant.

### Key and core genes validated

We first identified the key genes from hub genes by Logrank p-value<0.05 in overall survival and by Pr(>F)<0.05 in different pathological stages of patients with ovary cancer. Furthermore, we also analyzed patients with OSC for key genes in high-expression and low-expression groups at early stages (stages I+II) and advanced stages (stages III+IV). Simultaneously, we utilized GEPIA2 to confirm the expression of the key genes between OSC tissues and normal ovary tissues. According to the Hazard ratio and Logrank *p*-value of key genes in overall survival analysis for early stages (stages I+II) and advanced stages (stages III+IV), we defined the core genes from the key genes for early diagnosis in patients with OSC.

### EMT regulators selection and analysis

In order to identify the EMT-related regulatory genes in OSC development, we have compiled 15 EMT-related regulatory factors from published literatures [11–15, 18–20]. Then we systematically evaluated the expressed EMT-associated regulators and core genes in datasets GSE36668, GSE54388 and GSE69428, and then R ComplexHeatmap and dendextend packages were adopted to visualize them. Meanwhile, we used the R ggcorrplot package to estimate the correlation of core genes and EMT regulators. Then, Online database STRING was used to analysis the PPIs network and functional enrichment of core genes and EMT regulators. Next, we used another online tool NetworkAnalyst (http://www.networkanalyst.ca/) to visualize ovary specific PPIs network of core genes and EMT regulators. Furthermore, the online database cBioPortal for cancer genomics (https://www.cbioportal.org/) was used to analysis the genetic variation, mutation count and overall survival status related to the core genes and EMT regulators in OSC.

### GRN analysis of core genes and EMT regulators

We complied TF, core gene and EMT regulator co-network and analyzed the GRN by uploading the core gene and EMT regulator to NetworkAnalyst. The TF and gene target data were derived from the ENCODE (Encyclopedia of DNA Elements) ChIP-seq data. Only those objects with peak intensity signal <500 and the predicted regulatory potential score <1 could be selected using BETA Minus algorithm. Next, we complied miRNAs-core genes and EMT regulators co-network. The miRNA-gene interaction data validated by comprehensive experiments were collected from TarBase and miRTarBase. Soon after, we established the TF-miRNA integrated modulation network. Then, the integrated network was respectively visualized in Cytoscape to identify the core TF, EMT regulator and miRNA.

### Core genes, TF, EMT regulators and miRNA validation

We used Oncomine (http://www.oncomine.com) to evaluate the core genes on transcriptional level in multiple cancer types and relevant studies. The overall survival analyses related to core TF, EMT regulator and miRNA were performed using Kaplan-Meier Plotter. Then, we evaluated the significant core TF, EMT regulator expression among the GRN analysis and further validated using immunohistochemistry (IHC) from the Human Protein Atlas database (HPA, https://www.proteinatlas.org/). Simultaneously, we utilized the GEPIA2 to affirm the expression of core TF or EMT regulator between OSC tissues and normal ovary tissues.

### Experimental validation using quantitative real-time PCR in ovarian cancer lines

We explored the expression of core TF, EMT regulator and miRNA among different ovarian cancer cell lines in Cancer Cell Line Encyclopedia (CCLE). Then, ovarian cancer cell line (SKOV3) was obtained from American Type Culture Collection (Manassas, VA, USA) and maintained in Roswell Park Memorial Institute (RPMI)-1640 medium (Sigma-Aldrich, St Louis, MO, USA) with 10% fetal bovine serum (FBS) in a humidified atmosphere containing 5% CO_2_ at 37° C. Simultaneously, the human ovarian surface epithelium cells (HOSEC) were used as control. Total RNA was isolated by using a RNeasy Mini Kit (Qiagen) and cDNA was extracted by reverse transcription kit (Takara, Dalian, China). Gene expression was measured by qRT-PCR (Lightcycler96, Roche, Basel, Switzerland) using a SYBR Green™ Premix Ex Taq ™ II (Takara, Dalian, China) and following the manufacturer’s instructions. The primers used were shown in Supplementary Table 7.

### Statistical analyses

The significances of differences between two groups were analyzed using non-parametric test or *t*-test based on data distribution characteristics in Graphad Prism 8. The log-rank test was used to identify the differences in overall survival rate at different stages between low-expression and high-expression groups of hub genes using Kaplan-Meier Plots. Logrank *p*-value<0.05 was considered statistically significant in survival rate. Correlation analysis was calculated using R ggcorrplot package. All analyses were conducted using software R Studio 3.5.3. *P*-value < 0.05 was considered statistically significant.

## Supplementary Material

Supplementary Figures

Supplementary Table 1

Supplementary Table 2

Supplementary Table 3

Supplementary Tables 4, 5, 6 and 7

Supplementary Table 8

Supplementary Video 1

Supplementary Video 2

Supplementary Video 3
