# Supplementary material for: Identification of core genes for early diagnosis and the EMT modulation of ovarian serous cancer by bioinformatics perspective
Source: Aging (Albany NY). 2021 Jan 25;13(2):3112–45. doi: 10.18632/aging.202524 (PMC7880353; doi:10.18632/aging.202524)
Supplement: Supplementary Figures [file aging-13-202524-s001.pdf]

SUPPLEMENTARY FIGURES

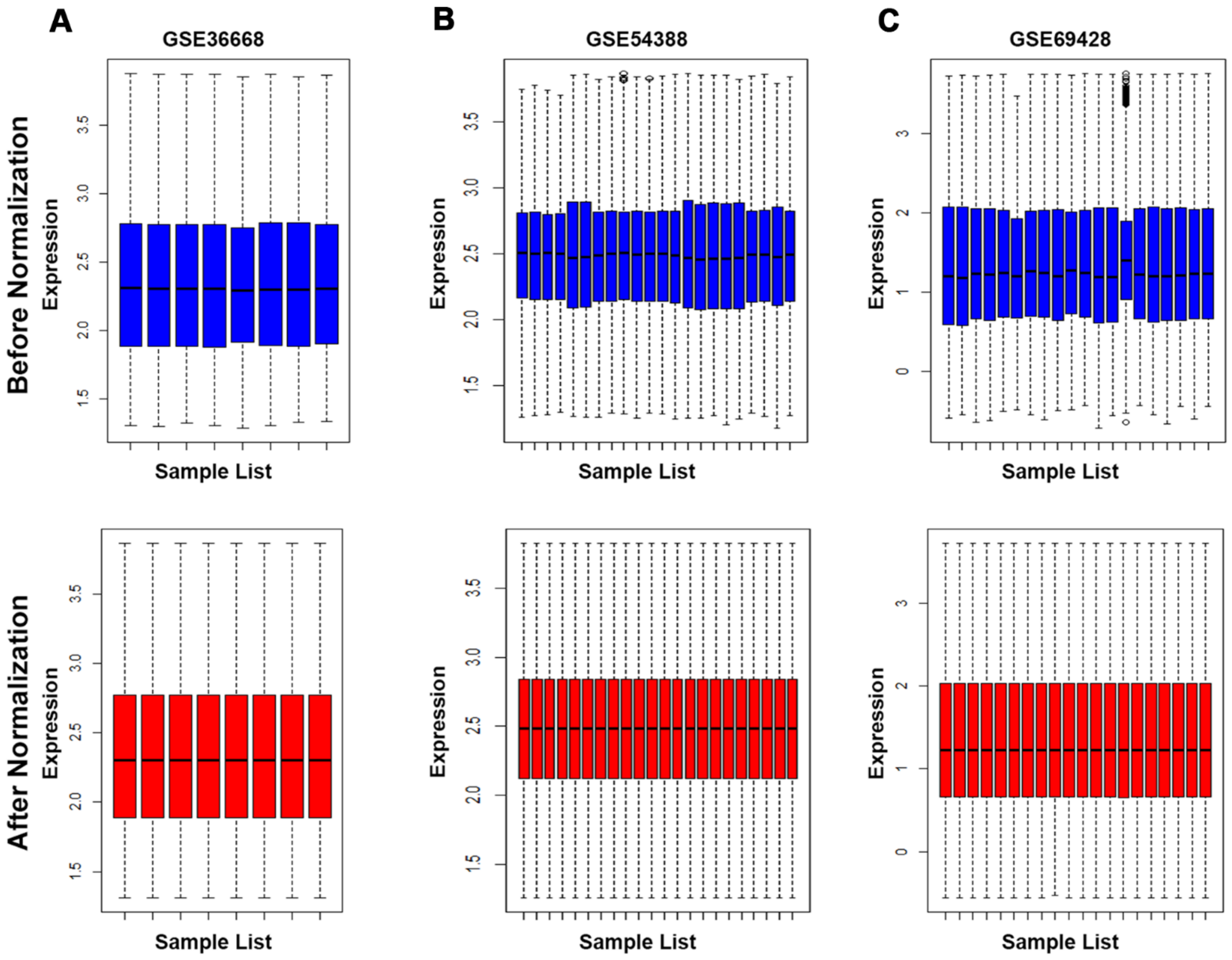

Supplementary Figure 1. Normalization of the raw data in GEO database. (**A**) Normalization of GSE36668 dataset. (**B**) Normalization of GSE54388 dataset. (**C**) Normalization of GSE69428 dataset. Blue represents raw data and red represents data after normalization.

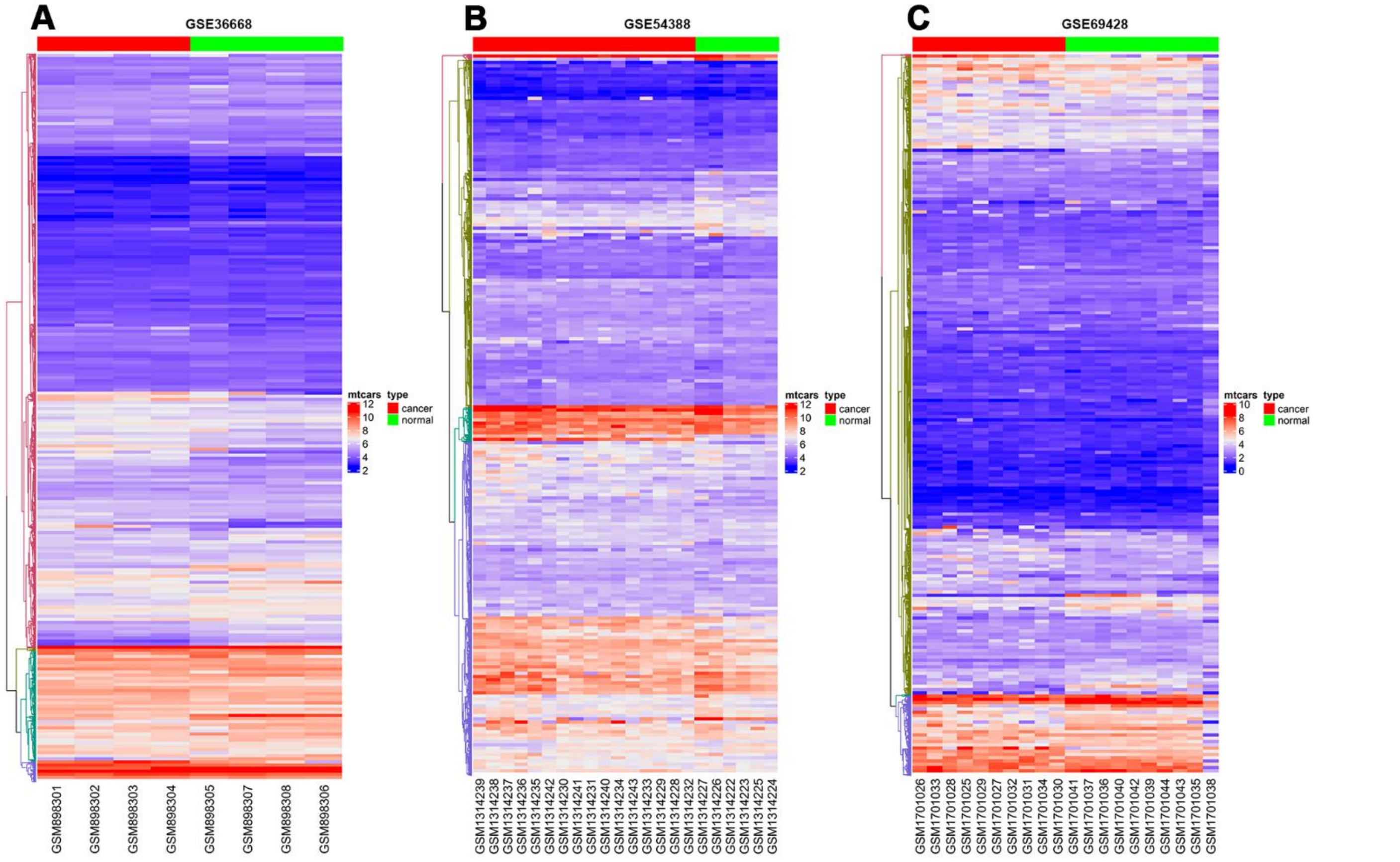

Supplementary Figure 2. The heatmap of gene expression in (**A**) GSE36668, (**B**) GSE54388 and (**C**) GSE69428. Red indicates a relatively high expression and blue indicates a relatively low expression.

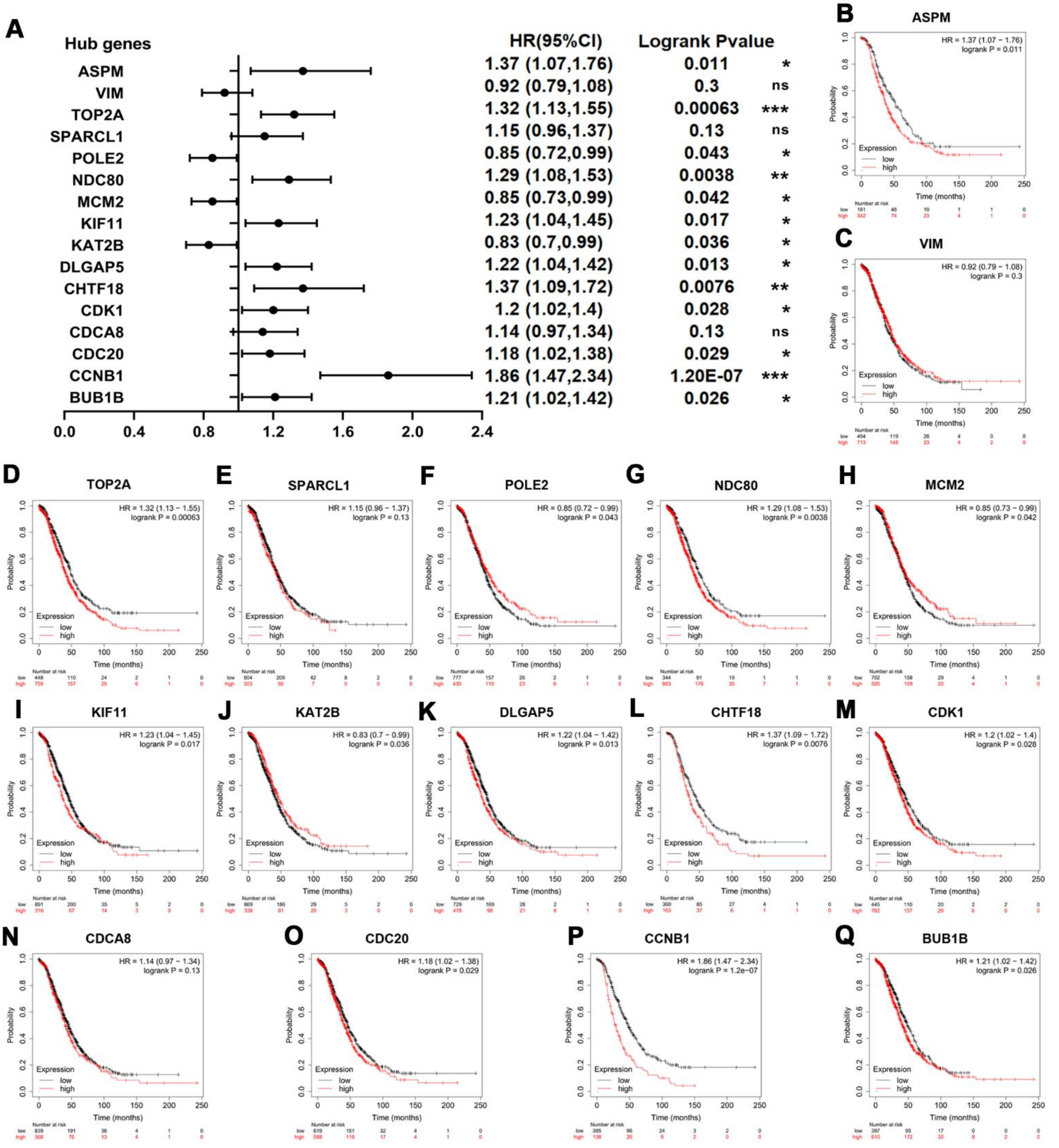

Supplementary Figure 3. Overall survival impact of hub genes on patients with OSC at all stages. (**A**) Survival prognosis forest map related to hub genes at all stages in OSC patients. (**B**–**Q**) Kaplan-Meier analysis for overall survival involved in the key genes expressed in OSC patients, covering (**B**) ASPM, (**C**) VIM, (**D**) TOP2A, (**E**) SPARCL1, (**F**) POLE2, (**G**) NDC80, (**H**) MCM2, (**I**) KIF11, (**J**) KAT2B, (**K**) DLGAP5, (**L**) CHTF18, (**M**) CDK1, (**N**) CDCA8, (**O**) CDC20, (**P**) CCNB1 and (**Q**) BUB1B. Logrank *p*-value<0.05 was considered statistically significant.

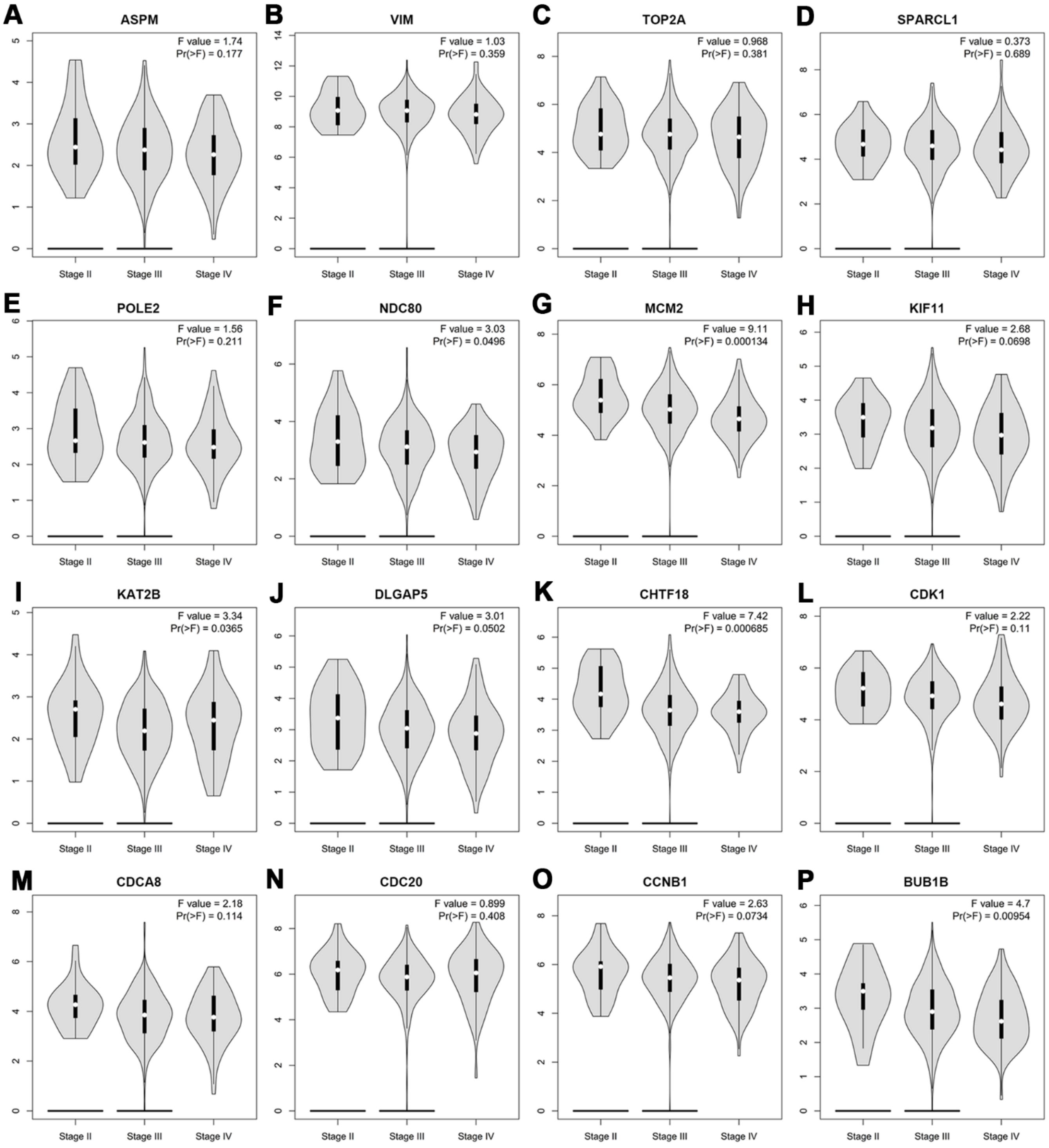

Supplementary Figure 4. The expression levels of hub genes in ovary cancer samples at different pathological stages. The hub genes (**A**) ASPM, (**B**) VIM, (**C**) TOP2A, (**D**) SPARCL1, (**E**) POLE2, (**F**) NDC80, (**G**) MCM2, (**H**) KIF11, (**I**) KAT2B, (**J**) DLGAP5, (**K**) CHTF18, (**L**) CDK1, (**M**) CDCA8, (**N**) CDC20, (**O**) CCNB1 and (**P**) BUB1B were analyzed respectively from the GEPIA2. ANOVA was performed to assess the statistical significance of variations. Pr(>F)<0.05 was considered statistically significant.

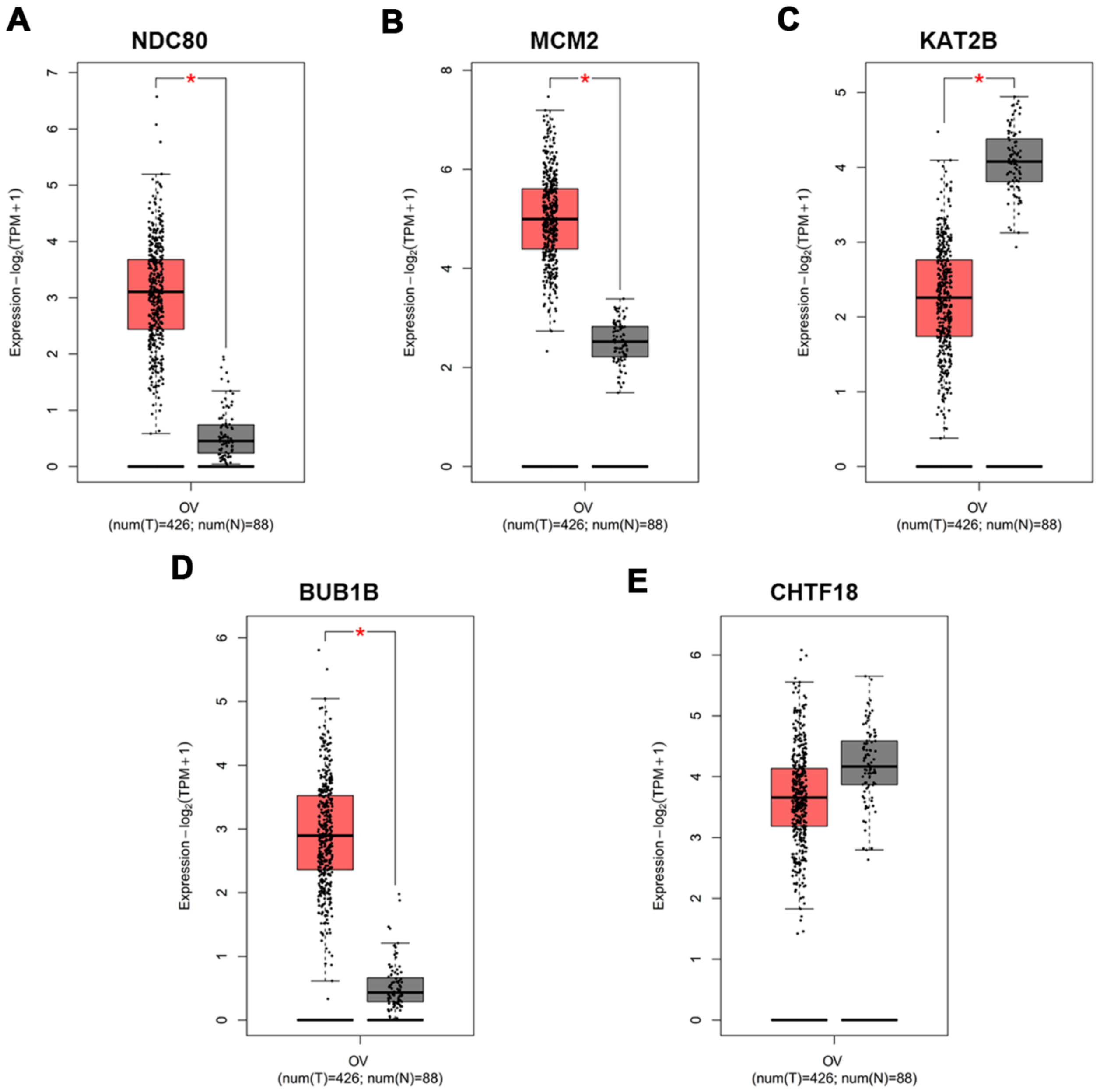

Supplementary Figure 5. The expression levels of key genes in ovary cancer samples and normal ovary samples. Based on TCGA and GTEx data from GEPIA2, we validated the expression levels of the key genes in ovarian cancer (n = 426, red) and normal tissues (n =88, gray). (**A**) NDC80, (**B**) MCM2, (**C**) KAT2B, (**D**) BUB1B and (**E**) CHTF18.

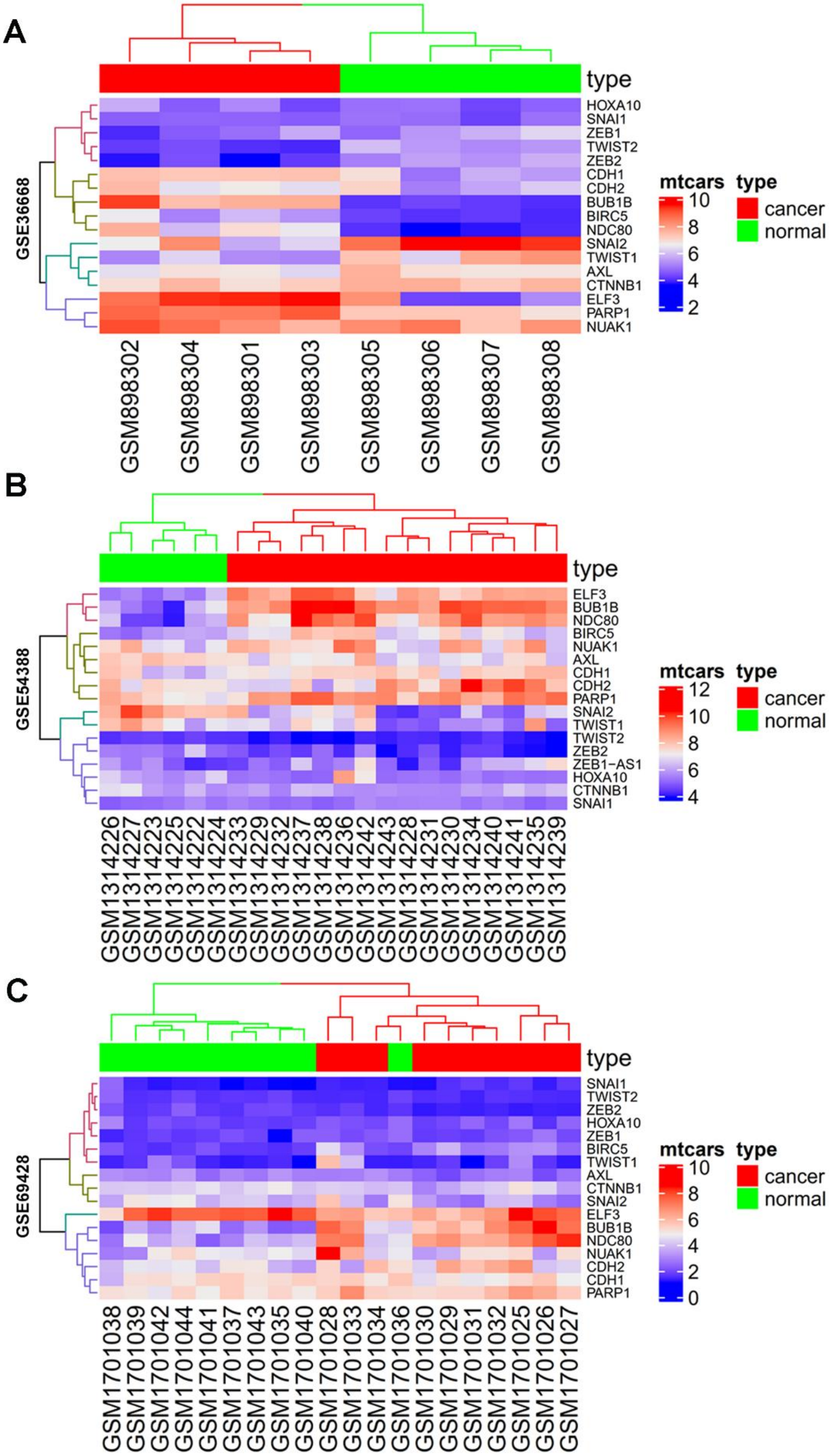

Supplementary Figure 6. The heatmap of differentially expressed core genes and EMT regulators between OSC tissues (red) and normal ovary tissues (green) in the datasets of (**A**) GSE36668, (**B**) GSE54388, (**C**) GSE69428. Blue indicates a relatively low expression and red indicates a relatively high expression.

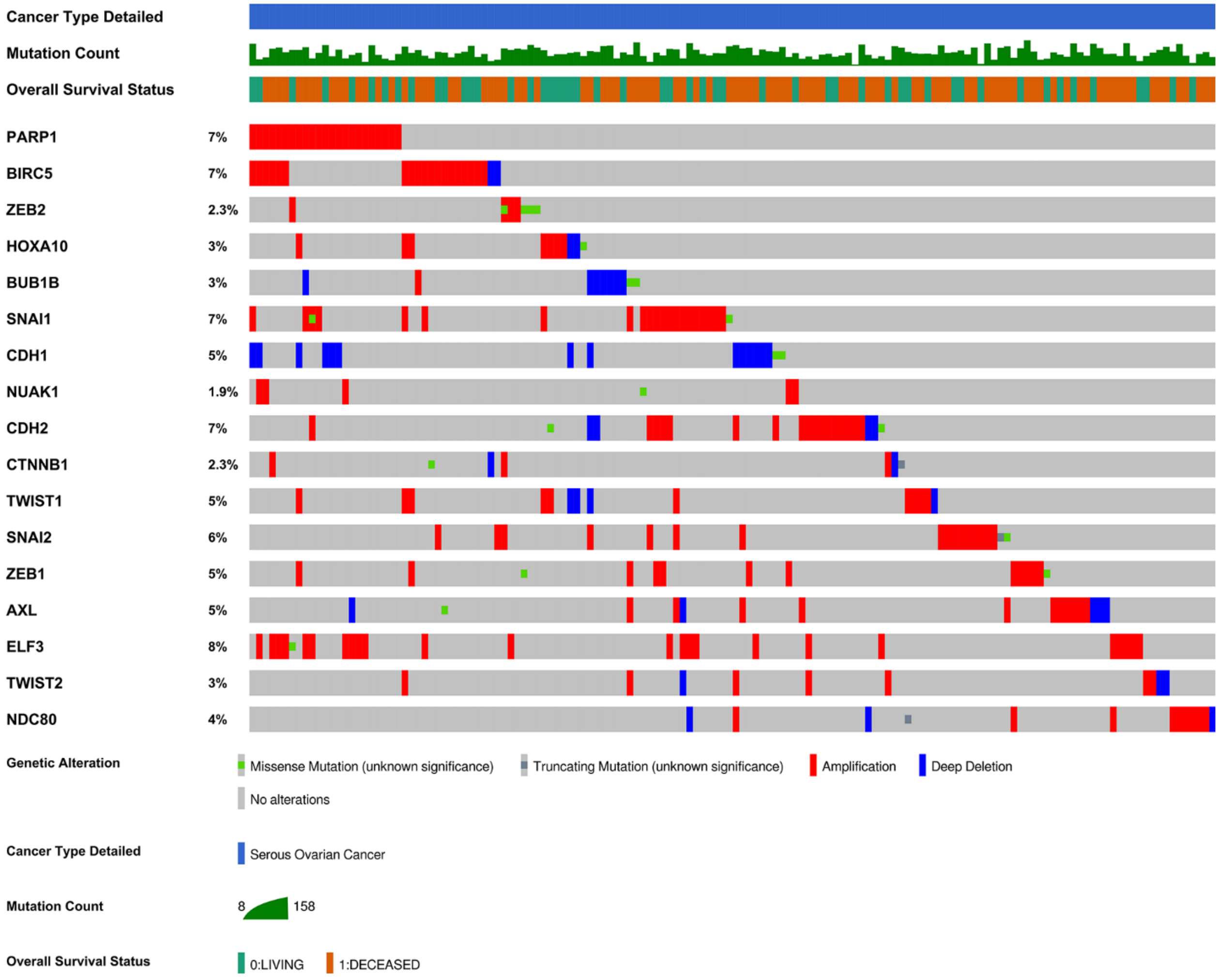

Supplementary Figure 7. The genetic variation, mutation count and overall survival status related to the core genes and EMT regulators are shown as a visual summary for a set of serous ovarian cancer samples from the TCGA database.

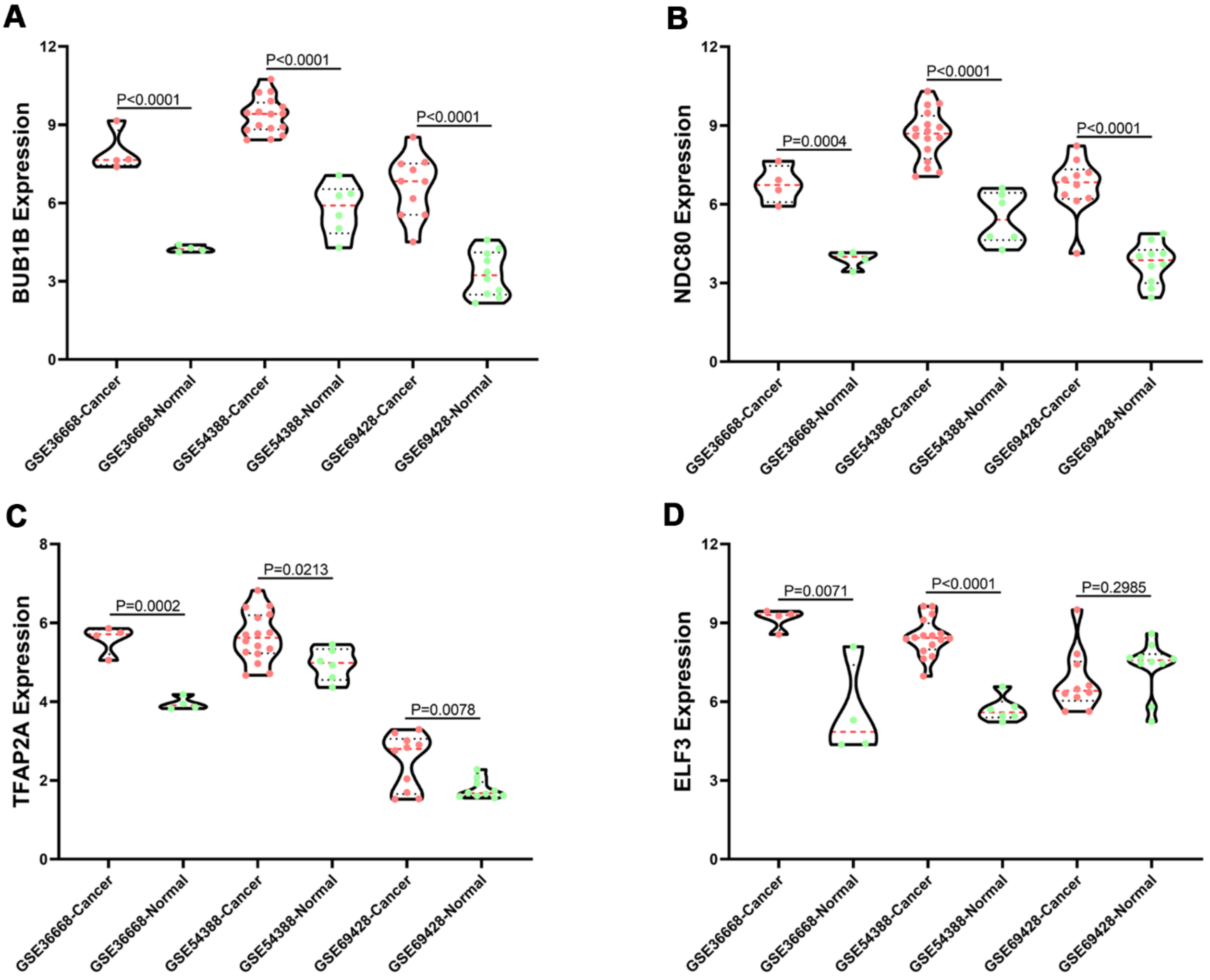

Supplementary Figure 8. Validation of core genes with relevant TF and EMT regulator in GSE36668, GSE54388 and GSE69428 datasets. The expression level analyses of (**A**) BUB1B, (**B**) NDC80, (**C**) TFAP2A, and (**D**) ELF3.

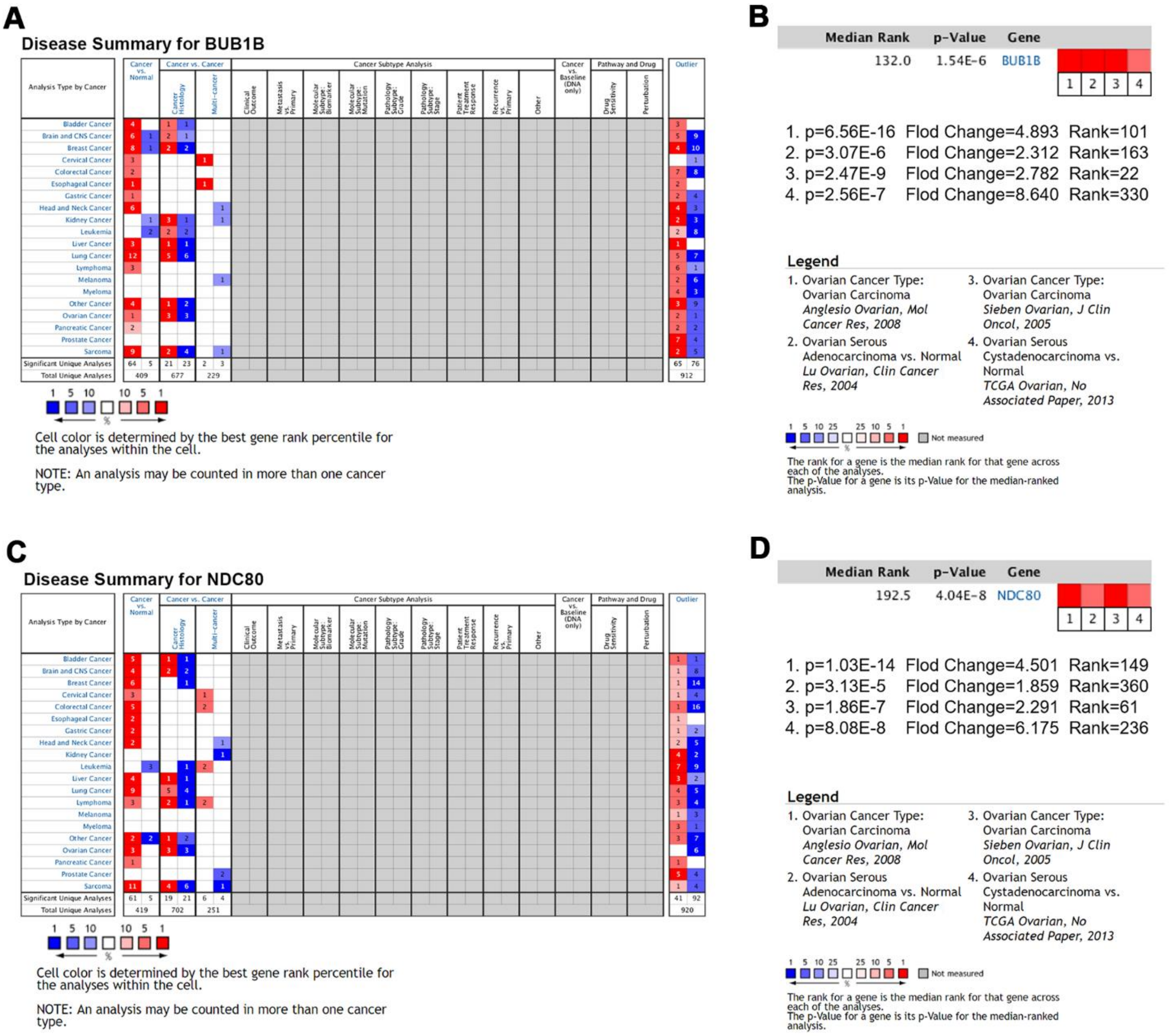

Supplementary Figure 9. Oncomine analysis of NDC80 and BUB1B expression in cancer tissues vs normal tissues. Validation of BUB1B (**A**) and NDC80 (**C**) on a transcriptional level in multiple cancer types and relevant studies. Heatmap represents BUB1B (**B**) and NDC80 (**D**) expression in ovarian cancer samples vs normal ovary samples. 1. ovarian carcinoma, Anglesio Ovarian, Mol Cancer Res, 2008; 2. ovarian carcinoma, Sieben Ovarian, J Clin Oncol, 2005; 3. Ovarian serous Adenocarcinoma vs Normal Lu Ovarian, Clin Cancer Res, 2004; 4. Ovarian serous Cystadenocarcinoma vs Normal TCGA Ovarian, No Associated Paper, 2013.

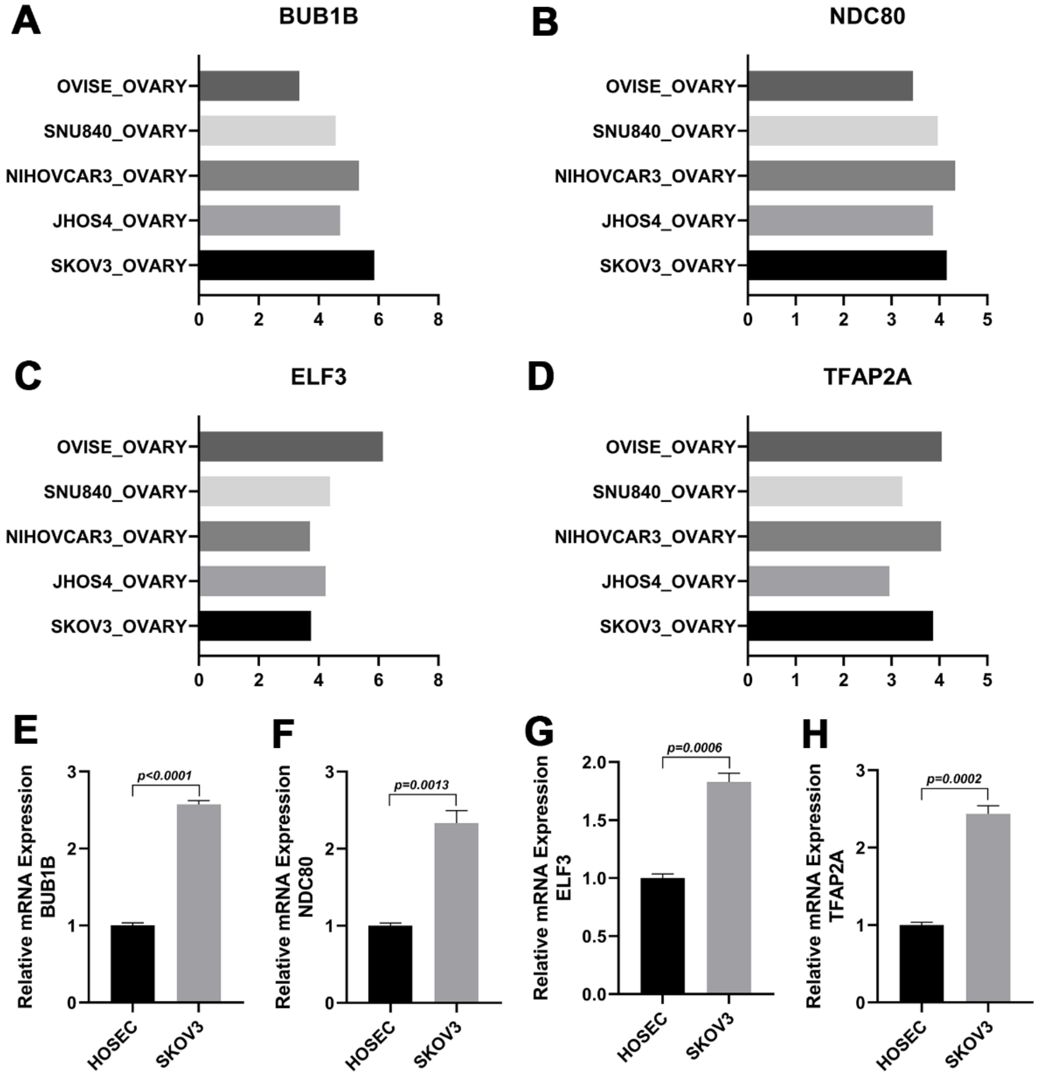

Supplementary Figure 10. The expression levels of core genes relevant with TF and EMT regulator in ovarian cancer cell lines. Analysis for BUB1B (**A**), NDC80 (**B**), ELF3 (**C**) and TFAP2A (**D**) from the CCLE in five different ovarian cancer cell lines. qRT-PCR assay was adopted to evaluate the expression of BUB1B (**E**), NDC80 (**F**), ELF3 (**G**) and TFAP2A (**H**) in SKOV3 compared with HOSEC as normal control.

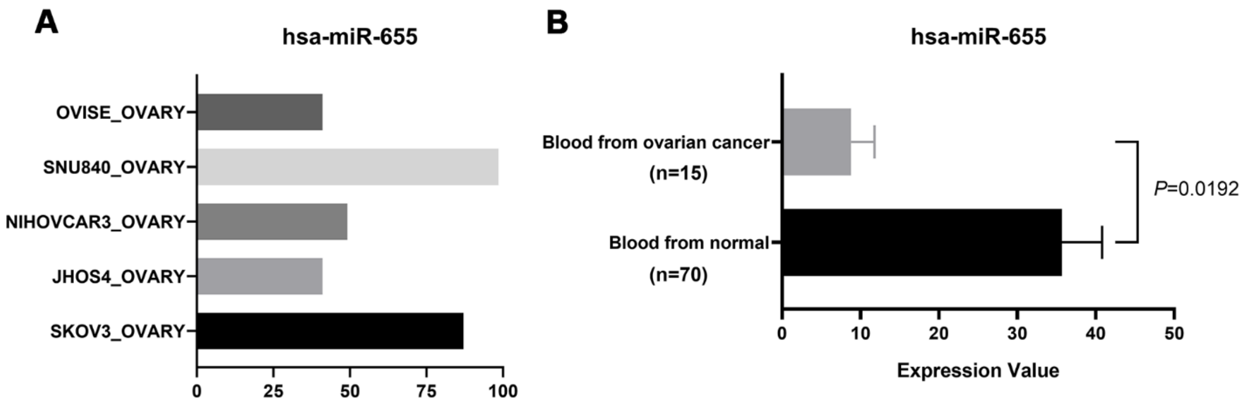

C

miRNA	logFC	SEQUENCE	Style
hsa-miR-200a	0.1878228	uaacacugucugguaacgaugu	Up
hsa-miR-204	-1.3228695	uucccuuugucauccuaugccu	Down
hsa-miR-214	0.4225062	acagcaggcacagacaggcagu	Up
hsa-miR-655	-1.9684933	auaauacaugguuaaccucuuu	Down
hsa-miR-124	0.7019605	uaaggcacgcggugaaugcc	Up
hsa-miR-145	0.1735157	guccaguuuucccaggaaucccu	Up
hsa-miR-25	0.0921893	cauugcacuugucucggucuga	Up
hsa-miR-32	-0.1025556	uauugcacauuacuaaguugca	Down
hsa-miR-34a	0.3019709	uggcagugucuuagcugguugu	Up
hsa-miR-369-3p	-1.1168674	aauaauacaugguugaucuuu	Down
hsa-miR-1	-1.0462092	uggaauguaaagaaguauguau	Down
hsa-miR-199a-5p	-0.1590583	cccaguguucagacuaccuguuc	Down
hsa-miR-369-5p	0.193202	agaucgaccguguuauauucgc	Up

Supplementary Figure 11. The expression level of hsa-miR-655. Analysis of hsa-miR-655 (**A**) from the CCLE in five different ovarian cancer cell lines. (**B**) The hsa-miR-655 expression and (**C**) the differential expression in blood miRNAs from ovarian cancer patients modulating core genes and EMT regulators compared with normal explored in GSE31568.
