# Supplementary material for: Identification of core genes for early diagnosis and the EMT modulation of ovarian serous cancer by bioinformatics perspective
Source: Aging (Albany NY). 2021 Jan 25;13(2):3112–45. doi: 10.18632/aging.202524 (PMC7880353; doi:10.18632/aging.202524)
Supplement: Supplementary Table 1 [file aging-13-202524-s002.pdf]

SUPPLEMENTARY TABLE

Supplementary Table 1. Details of ovarian serous cancer associated microarray datasets from GEO database.

Reference	sample	GEO	Platform	Normal ovary	Ovarian serous cancer
Elgaaen et al (2012)	Ovarian	GSE36668	GPL570	4	4
Yeung et al (2017)	Ovarian	GSE54388	GPL570	6	16
Yamamoto et al (2015)	Ovarian	GSE69428	GPL570	10	10
