# Supplementary material for: Identification of core genes for early diagnosis and the EMT modulation of ovarian serous cancer by bioinformatics perspective
Source: Aging (Albany NY). 2021 Jan 25;13(2):3112–45. doi: 10.18632/aging.202524 (PMC7880353; doi:10.18632/aging.202524)
Supplement: Supplementary Tables 4, 5, 6 and 7 [file aging-13-202524-s005.pdf]

SUPPLEMENTARY TABLES

Supplementary Table 4. Module analysis of co-DEGs using MCODE of Cytoscope.

MCODE_Cluster	MCODE_Node_Status	MCODE_Score	name	TopologicalCoefficient
Cluster 1	Clustered	21.32307692	CDK1	0.24610988
Cluster 1	Clustered	21.32307692	CCNB1	0.28399209
Cluster 1	Clustered	21.32307692	CDC20	0.29765066
Cluster 1	Clustered	21.32307692	KIF11	0.32624113
Cluster 1	Clustered	21.32307692	TOP2A	0.33646245
Cluster 1	Clustered	21.32307692	CDCA8	0.34704034
Cluster 1	Clustered	21.32307692	BUB1B	0.36419753
**Cluster 1**	**Seed**	**21.32307692**	**ASPM**	**0.36976744**
Cluster 1	Clustered	21.32307692	DLGAP5	0.38181271
Cluster 1	Clustered	21.32307692	NDC80	0.35257453
Cluster 1	Clustered	21.32307692	KIF2C	0.40125
Cluster 1	Clustered	21.32307692	CENPF	0.40493421
Cluster 1	Clustered	21.32307692	TTK	0.40350877
Cluster 1	Clustered	21.32307692	KIF20A	0.40773229
Cluster 1	Clustered	18.8034188	RRM2	0.34723854
Cluster 1	Clustered	21.32307692	NUSAP1	0.41392318
Cluster 1	Clustered	20.51	NCAPG	0.40590278
Cluster 1	Clustered	20.37	TPX2	0.40785714
Cluster 1	Clustered	20.37	CEP55	0.41801471
Cluster 1	Clustered	20.69565217	UBE2C	0.42312009
Cluster 1	Clustered	19.98290598	PBK	0.44112903
Cluster 1	Clustered	18	ESPL1	0.39016897
Cluster 1	Clustered	20.44	KIF4A	0.45512821
Cluster 1	Clustered	19.60474308	KIF15	0.44137931
Cluster 1	Clustered	19.49275362	SPAG5	0.45767196
Cluster 1	Clustered	20.69565217	MELK	0.46383929
Cluster 1	Clustered	18.90952381	NUF2	0.45238095
Cluster 1	Clustered	19.76284585	KIF23	0.43717728
Cluster 1	Clustered	17.81052632	PRC1	0.41557223
Cluster 1	Clustered	18.81904762	PTTG1	0.46615385
Cluster 1	Clustered	18.90952381	MKI67	0.48839662
Cluster 1	Clustered	17.90526316	CDCA3	0.50185185
Cluster 2	Clustered	14	KNTC1	0.48070175
Cluster 2	Clustered	14	CENPU	0.47387387
Cluster 2	Clustered	14	ZWINT	0.45855856
Cluster 2	Clustered	14	RCC2	0.48841699
Cluster 2	Clustered	14	CDCA5	0.48841699
Cluster 3	Clustered	5.727272727	PCNA	0.23539618
Cluster 3	Clustered	5.785714286	MCM10	0.22094361
Cluster 3	Clustered	5.5	FEN1	0.31566265
Cluster 3	Clustered	5	DTL	0.34993614
Cluster 3	Clustered	5	DNA2	0.40613027
Cluster 3	Clustered	5	TIMELESS	0.41666667
Cluster 3	Clustered	5.785714286	ORC6	0.35030864
Cluster 3	Clustered	5	RMI2	0.44387755

**Cluster 3**	**Seed**	**6**	**CHTF18**	**0.53571429**
Cluster 3	Clustered	5.785714286	CDC7	0.30892857
Cluster 3	Clustered	5	RAD51AP1	0.52857143
Cluster 4	Clustered	7.418181818	MCM3	0.26919476
Cluster 4	Clustered	8	MCM7	0.26763285
**Cluster 4**	**Seed**	**8**	**MCM2**	**0.25791624**
Cluster 5	Clustered	3.771428571	RFC4	0.19430761
**Cluster 5**	**Seed**	**4.581818182**	**POLE2**	**0.2029321**
Cluster 5	Clustered	4	ISG15	0.50641026
Cluster 6	Clustered	2	LAMC1	0.66666667
**Cluster 6**	**Seed**	**2**	**SPARCL1**	**0.83333333**
Cluster 6	Clustered	2	STC2	0.83333333
**Cluster 7**	**Seed**	**2**	**VIM**	**1**
Cluster 7	Clustered	2	TNNI3	1
Cluster 7	Clustered	2	TNNT1	1
**Cluster 8**	**Seed**	**2**	**KAT2B**	**0.43333333**
Cluster 8	Clustered	2	E2F3	0.53333333
Cluster 8	Clustered	2	MEF2C	0.75
Cluster 9	Clustered	2	GAPDH	1
Cluster 9	Clustered	2	TPI1	1
**Cluster 9**	**seed**	**2**	**GPI**	**1**

Supplementary Table 5. Hub genes evaluated by PPI network analysis Degree>40 or the seed genes in MOCODE analysis of Cytoscape.

Entrez ID	Gene symbol	Description	Alternate names	Chromosome	Map location
63922	CHTF18	chromosome transmission fidelity factor 18	C16orf41|C321D2.2|C321D2.3|C321D2.4|CHL12|Ctf18|RUVBL	16	16p13.3
4171	MCM2	minichromosome maintenance complex component 2	BM28|CCNL1|CDCL1|D3S3194|MITOTIN|cdc19	3	3q21
5427	POLE2	polymerase (DNA directed), epsilon 2, accessory subunit	DPE2	14	14q21-q22
8404	SPARCL1	SPARC-like 1 (hevin)	MAST 9|MAST9|PIG33|SC1	4	4q22.1
7431	VIM	vimentin	CTRCT30|HEL113	10	10p13
8850	KAT2B	K(lysine) acetyltransferase 2B	CAF|P/CAF|PCAF	3	3p24
983	CDK1	cyclin-dependent kinase 1	CDC2|CDC28A|P34CDC2	10	10q21.1
891	CCNB1	cyclin B1	CCNB	5	5q12
991	CDC20	cell division cycle 20	CDC20A|bA276H19.3|p55CDC	1	1p34.1
3832	KIF11	kinesin family member 11	EG5|HKSP|KNSL1|MCLMR|TRIP5	10	10q24.1
7153	TOP2A	topoisomerase (DNA) II alpha 170kDa	TOP2|TP2A	17	17q21-q22
55143	CDCA8	cell division cycle associated 8	BOR|BOREALIN|DasraB|MESRGP	1	1p34.3
701	BUB1B	BUB1 mitotic checkpoint serine/threonine kinase B	BUB1beta|BUBR1|Bub1A|MAD3L|MVA1|SSK1|hBUBR1	15	15q15
259266	ASPM	asp (abnormal spindle) homolog, microcephaly associated (Drosophila)	ASP|Calmbp1|MCPH5	1	1q31
9787	DLGAP5	discs, large (Drosophila) homolog-associated protein 5	DLG7|HURP	14	14q22.3
10403	NDC80	NDC80 kinetochore complex component	HEC|HEC1|HsHec1|KNTC2|TID3|hsNDC80	18	18p11.32

Supplementary Table 6. KEGG enrichment of core genes and EMT regulators.

Pathway ID	Description	Count in gene set	False Discovery Rate
hsa04520	Adherens junction	4 of 71	1.77E-05
hsa04390	Hipposignaling pathway	3 of 152	0.0059
hsa05216	Thyroid cancer	2 of 37	0.0067
hsa05205	Proteoglycans in cancer	3 of 195	0.0067
hsa05213	Endometrial cancer	2 of 58	0.008
hsa05130	Pathogenic Escherichia coli infection	2 of 53	0.008
hsa05412	Arrhythmogenic right ventricular cardiomyopathy	2 of 72	0.0103
hsa05100	bacterial invasion of Epithelial cells	2 of 72	0.0103
hsa05215	Prostate cancer	2 of 97	0.0142
hsa04514	cell adherion molecules(CAMs)	2 of 139	0.0254
hsa05226	Gastric cancer	2 of 147	0.0257
hsa05206	MicroRNAs in cancer	2 of 149	0.0257
hsa05202	Transcriptional misregulation in cancer	2 of 169	0.0284
hsa04015	Rap1 signaling pathway	2 of 203	0.0373

Supplementary Table 7. Primers used for the real-time PCR.

Gene	Primers	Sequences (5'-3')
β-actin	Forward	CCACGAAACTACCTTCAACTCC
	Reverse	GTGATCTCCTTCTGCATCCTGT
ELF3	Forward	GCAAACACCCTGGACTTGAC
	Reverse	CTGTCCTCTCATCACGGTGC
TFAP2A	Forward	ATATCCGTTCACGCCGATCC
	Reverse	CCTCGCAGTCCTCGTACTTG
BUB1B	Forward	GGGATGGGTCCTTCTGGAAA
	Reverse	GGCATTCAGAATCCGCACAA
NDC80	Forward	CCTGGTGTTTTGATGACCGC
	Reverse	TCCAGTTTCCTGACACGACC
